# Variable blood processing procedures contribute to plasma proteomic variability

**DOI:** 10.1186/s12014-021-09311-3

**Published:** 2021-01-19

**Authors:** Patrick Halvey, Victor Farutin, Laura Koppes, Nur Sibel Gunay, Dimitrios A. Pappas, Anthony M. Manning, Ishan Capila

**Affiliations:** 1 Momenta Pharmaceuticals Inc, 301 Binney Street, Cambridge, MA 02142 USA; 2 Department of Medicine, Division of Rheumatology, Columbia University School of Medicine, New York, NY USA

**Keywords:** Plasma proteomics, Cohort study, LC–MS/MS, Sample processing, Preanalytical variability

## Abstract

**Background:**

Plasma is a potentially rich source of protein biomarkers for disease progression and drug response. Large multi-center studies are often carried out to increase the number of samples analyzed in a given study. This may increase the chances of variation in blood processing and handling, leading to altered proteomic results. This study evaluates the impact of blood processing variation on LC–MS/MS proteomic analysis of plasma.

**Methods:**

Initially two batches of patient plasma samples (120 and 204 samples, respectively) were analyzed using LC–MS/MS shotgun proteomics. Follow-up experiments were designed and carried out on healthy donor blood in order to examine the effects of different centrifugation conditions, length of delay until first centrifugation, storage temperature and anticoagulant type on results from shotgun proteomics.

**Results:**

Variable levels of intracellular proteins were observed in subsets of patient plasma samples from the initial batches analyzed. This observation correlated strongly with the site of collection, implicating variability in blood processing procedures. Results from the healthy donor blood analysis did not demonstrate a significant impact of centrifugation conditions to plasma proteome variation. The time delay until first centrifugation had a major impact on variability, while storage temperature and anticoagulant showed less pronounced but still significant effects. The intracellular proteins associated with study site effect in patient plasma samples were significantly altered by delayed processing also.

**Conclusions:**

Variable blood processing procedures contribute significantly to plasma proteomic variation and may give rise to increased intracellular proteins in plasma. Accounting for these effects can be important both at study design and data analysis stages. This understanding will be valuable to incorporate in the planning of protein-based biomarker discovery efforts in the future.

## Background

Blood plasma is a readily accessible biofluid which may be used in biomarker discovery [1]. In particular, proteins (or protein fragments) in circulation may be associated with disease susceptibility, disease activity, or drug response. Frozen plasma can be stored for decades with minimal degradation of proteins over time, making it an excellent choice for retrospective biomarker studies [2]. Discovery proteomic platforms seek to use large collections of patient plasma samples to identify the key proteins which correlate with some aspect of disease or therapeutic response. Acquiring sufficiently large numbers of samples often requires plasma to be collected from multiple centers. Despite the use of standardized blood collection and processing protocols, certain preanalytical variables may contribute to proteomic variation across collection sites. Increasing evidence suggests that deviations from best practices in blood processing may contribute to variable proteomic results [3].

Previous studies have established that preanalytical variables can impact both individual protein measurements and proteome-wide measurements. Variables include centrifugation conditions, time till first centrifugation of blood, choice of anti-coagulant, storage length and number of freeze/thaw cycles. Previous studies have demonstrated the impact on plasma concentrations of selected proteins as measured by ELISA of multiple freeze/thaw cycles [4] and platelet enrichment, thus implicating centrifugation as a key variable [5]. Additionally, an evaluation of a broad panel of plasma analytes has demonstrated effects of storage temperature and duration to the first centrifugation on measured levels of several additional proteins in plasma that can be assessed by clinical laboratory instruments [6].

LC–MS/MS-based discovery proteomics have been used to show the proteome-wide effects of preanalytical variables. Interestingly, several studies have failed to observe major protein changes in blood stored for prolonged periods prior to first centrifugation [7–9]. However, in these studies samples were pooled together prior to LC–MS/MS analysis which may prevent individual protein changes from being detected. An additional study found that after a 96 h delay 41 and 83 proteins showed significant changes at room temperature and 37 °C, respectively [2]. Crucially, no sample pooling was carried out in this study. Previous proteome-wide analysis of preanalytical variables have typically used blood from healthy volunteers, and few attempts have been made to link these observations with proteomics data from large multi-center patient studies.

Here we carried out LC–MS/MS shotgun proteomics on two sets of patient samples (120 and 204 samples, respectively) drawn from 30 different collection sites. We observed changes in protein expression which appeared to be related to blood processing conditions. In particular, we noted increased intracellular protein levels in plasma samples collected at specific locations. To investigate this phenomenon further, we then examined the impact of pre-analytical variables in healthy volunteer samples. Variables included centrifugation conditions, time delay to first centrifugation, blood storage temperature and anti-coagulant. We found that specific variables are likely to contribute strongly to the observation of the increase in intracellular protein levels in patient plasma samples.

## Methods

### Blood collection and processing for patient samples

Blood was collected from patients at multiple collection centers across the United States as part of a prospective non-randomized “real world” comparative effectiveness study by a national patient registry with study design, enrollment criteria and quality control procedures as previously published [10]. For the purposes of proteomics analyses for a balanced by clinical status randomized subset of patients two samples were selected from each subject at two different timepoints. Representative patient demographics are provided in Additional file 1: Table S1. Blood was drawn by venipuncture into 7 mL sodium heparin glass tubes until vacuum was exhausted and blood ceased to flow. Per sample collection SOPs, the tubes were inverted 8–10 times and blood was immediately centrifuged at 1300×*g* for 10 min. Plasma was carefully pipetted off and aliquoted into 2 mL cryogenic vials (Corning). Plasma samples were frozen at − 20 °C until they could be shipped (within one month after collection) to a central repository for long-term storage (at − 20 °C) and redistribution. Samples were shipped to Momenta Pharmaceuticals on dry ice, thawed, aliquoted and stored at − 80 °C until ready for proteomic analysis.

### Blood collection for healthy volunteer experiments

For internal experiments with healthy volunteers blood was collected by a trained phlebotomist. Blood was collected into 10 mL BD vacutainer tubes containing either K2-EDTA or lithium heparin (BD, Franklin Lakes, NJ). For the three single donor pilot studies blood processing variables were evaluated as summarized by the Fig. 2a–c. Briefly, for single donor centrifugation experiments blood was spun at 1000×*g* or 2000×*g* for 10 min in a swing bucket centrifuge (Sorvall Legend XFR, Thermo Fisher Scientific) at room temperature. The centrifuge brake was set either to maximum deceleration at the end of the run or was not applied at all. After the first centrifugation step plasma was carefully pipetted off and transferred to a 1.7 mL Eppendorf tube for storage or to 15 mL polypropylene tube (Corning) for a second centrifugation step. The second centrifugation step was carried out as described above. Afterwards, all plasma samples were stored at − 80 °C. For both the temperature/time single donor study and the time/anticoagulant type single donor study blood was spun twice for 10 min at 1500×*g* with maximum brake deceleration applied.

For the temperature and time single donor study, blood was collected into EDTA tubes. For time and anticoagulant type single donor study until the first centrifugation blood was stored at ambient temperature. For multi-donor experiments blood from four healthy volunteers was collected as described above and held for varying lengths of time before the first centrifugation at either room temperature or 4 °C. Blood was centrifuged twice at 1500×*g* for 10 min with maximum brake deceleration applied. Plasma samples were stored at − 80 °C.

### Immunodepletion and protein digestion

All sample preparation reagents were purchased from Sigma-Aldrich (St. Louis, MO) unless otherwise stated. Plasma samples were thawed at room temperature and filtered using 0.22 µm spin filters (Agilent Technologies, Santa Clara, CA). Samples were subjected to depletion of high abundant proteins using a multiple affinity removal system (MARS-14) immunodepletion column (4.6 × 100 mm, Agilent Technologies). A HPLC system with autosampler, quaternary pump, UV detector and fraction collector (Agilent Technologies) was used to separate plasma proteins on the MARS-14 column in accordance with the manufacturer’s protocol. The low abundant protein fraction was buffer exchanged into 50 mM ammonium bicarbonate using 5 kDa MWCO spin concentrators (Agilent Technologies) and desalted with 7 kDa MWCO Zeba spin columns (Thermo Fisher Scientific). Protein concentrations were measured using the BCA assay (Thermo-Fisher Scientific). For all samples 50 µg of immunodepleted proteins were digested. Proteins were reduced with 100 mM dithiothreitol (DTT) and 80 mM tris (2-carboxyethyl)phosphine (TCEP) for 30 min at 60 °C and alkylated with 200 mM iodoacetamide for 20 min at room temperature. Volumes were brought up to 500 µL with 50 mM ammonium bicarbonate and trypsin/lys-C enzyme mix (Promega, Madison, WI) was added (25:1, protein:enzyme ratio). Samples were digested for 16 h at 37 °C, frozen and lyophilized. Sample clean-up was performed on an Oasis HLB plate (5 mg sorbent, 30 µm particle size) (Waters corporation, Milford, MA). Peptides were dried under vacuum and resuspended in 50 µL 0.1% formic acid and stored at − 80 °C prior to LC–MS/MS analysis.

### LC–MS/MS analysis and database searching

All LC–MS/MS consumables including solvents and columns were purchased from Thermo-Fisher Scientific unless otherwise stated. For LC–MS/MS shotgun proteomic analysis of peptide mixtures, separations were carried out using an Ultimate 3000 RSLC nano system (Thermo-Fisher Scientific). Peptides were loaded onto an Acclaim PepMap RSLC Nano trap column (5 µm particle size, 20 mm × 100 µm) at 5 µL min^−1^ flow rate, using a loading solvent of 0.1% trifluoroacetic acid. Peptides were resolved on an EASY-Spray Acclaim PepMap RSLC C18, 2 µm particle, 100 Å pore size, 0.075 × 250 mm column thermostatically controlled at 50 °C with a 300 nL min^−1^ flow rate and linear gradient from 1 to 30% acetonitrile containing 0.1% (v/v) formic acid for a total duration of 150 min. Solvent A contained 5% DMSO in order to increase sensitivity during the early portion of the chromatogram [11]. After the gradient portion of the chromatogram the column was washed with 99% acetonitrile for 20 min and equilibrated with 1% acetonitrile for 30 min. MS analyses were performed on an Orbitrap Velos Pro in the positive-ion mode using an EASY-Spray nano-source (Thermo-Fisher Scientific). The instrument was operated with the spray voltage of 1.8 kV, an ion transfer capillary temperature of 275 °C and S lens RF level of 70%. One high resolution FTMS scan of 60,000 resolution including 1 micro scan with maximum injection time of 50 ms was followed by 24 dependent MS/MS scans with maximum injection time of 100 ms. Dependent MS/MS scans were performed using an isolation width of 2.0 m/z around parent ions. The isolated multiple charged ions (2, 3, 4 or higher) were activated using the CID parameters of 10 ms activation time and 35% normalized collision energy. Data-dependent scan dynamic exclusion was enabled. The dynamic exclusion settings were as follows: repeat count 1, repeat duration 20 s, exclusion list size 500, exclusion duration 30 s, exclusion mass width low 0.5, exclusion mass width high 1.5, expiration count 2 and expiration S/N threshold 2.

Samples were randomized into batches of 20 for LC–MS/MS analysis. A plasma quality control sample was included in every batch (Sigma-Aldrich) and was used to assess performance across each study. The control plasma sample was subjected to an identical workflow to that described above and was used to assess variability in digestion efficiency, number of peptide-spectrum matches (PSMs) and number of proteins identified. On average, the following variability metrics have been observed across plasma quality control samples: digestion efficiency—1% CV, #PSMs—3.3% CV and total #proteins—5.4% CV. A HeLa cell digest (Thermo-Fisher) was also run at regular intervals to monitor mass accuracy, chromatographic performance and instrument sensitivity over time.

Proteome Discoverer (v1.4, Thermo-Fisher) was used to generate PSM counts from RAW files. Each RAW file was run through a workflow which included spectrum filtering, protein database searching using Sequest-HT and peptide-level FDR calculation. For MS1 spectrum filtering a signal/noise threshold of 1.5 was used. The human Uniprot database (protein isoforms included) was used. Mass tolerances of 10 ppm and 0.8 Da were used for precursor ions and fragment ions, respectively. The minimum peptide length was 6 amino acids with a maximum of two missed tryptic cleavages. Peptide dynamic modifications included N-terminal acetylation (+ 42.011 Da) and methionine oxidation (+ 15.995 Da). Carbamidomethylation of cysteine (+ 57.021 Da) was included as a static modification. For peptide scoring the maximum delta Cn was set to 0.05. Protein grouping, peptide grouping and maximum parsimony were enabled. A 5% peptide-level false discovery rate was applied using the target decoy PSM validator node within Proteome Discoverer. A multi-consensus report was prepared for each experimental group and protein group-level results were exported for normalization and statistical analysis (Additional file 2: Tables S11–S16). Raw data from designed experiments has been deposited to MassIVE repository (University of California San Diego, CA, USA, http://massive.ucsd.edu) with accession numbers MSV000085763, MSV000085850, MSV000085799 and MSV000085814. In order to generate a set of high confidence protein identifications a minimum of two peptides per protein and at least one proteotypic peptide per protein were required. Furthermore, within a given study proteins with less than an average of one PSM per RAW file were excluded. For analysis of protein expression levels we used spectral counting, a label-free method for quantitation of MS-derived proteomics data. Spectral counting has been previously shown to compare favorably with label-based approaches such as iTRAQ [12].

Additionally, the identified proteins in each sample group were compared to the set of proteins in the human plasma build “Plasma Non-Glyco 2017-04—Mapping 2017-05-12” of PeptideAtlas [13]. Proteins were then categorized as “canonical” and “not observed” in accordance with the annotations used by PeptideAtlas as shown in Additional file 1: Section S1 and summarized below.

### Data normalization and statistical analysis

Statistical data analysis including pre-processing and normalization of PSM counts was performed using R/Bioconductor statistical software [14, 15]. Dissimilarities between samples within each translational study collection were calculated as one complement of rank based (Spearman) correlation between protein abundances in each sample. This dissimilarity measure is invariant to the choice of sample normalization as long as it does not alter rank order of protein abundances within the sample. R implementation (cmdscale) of classical multidimensional scaling (aka principal coordinates analysis [16, 17]) of the resulting dissimilarity matrix was used to visualize dissimilarities between samples in the two translational study collections. Correlations of average protein levels across translational study sites relied on the ranks of corresponding PSM counts within each sample. Analysis of differences in protein levels across experimental conditions studied in designed experiments (Additional file 3: Tables S7–S10) was performed within limma-voom methodology relying on default settings available therein [18, 19]. In brief, protein counts were normalized to their sample total and log transformed prior to the accounting for mean–variance trend and linear model fitting. Statistical significance of the observed associations between protein levels and experimental conditions has been estimated as Benjamini–Hochberg false discovery rate (BH-FDR) to account for multiple tests within each study [20]. Additional details including data pre-processing prior to statistical analyses can be found in Additional file 1: Section S1.

## Results

### Observation of intracellular proteins in patient plasma samples

As part of a biomarker discovery effort we carried out shotgun proteomic analysis of two separate sample batches from a national patient registry. For the purposes of the study two samples were collected for each patient, one at the time of subject enrollment in the study and one at a later timepoint. A total of 120 (60 patients) and 204 (102 patients) plasma samples were analyzed in study one and study two, respectively. The samples for both studies were drawn from 30 collection centers across the United States of which 20 were common to the two studies. For each batch samples were randomized and processed in groups of 20 (immunodepletion, digestion, LC–MS/MS shotgun proteomics). The median count of proteins detected per sample and corresponding interquartile range (IQR) was 351 (IQR = 20) and 373 (IQR = 28) for the first and second batches respectively. The median percentage of proteins that are “canonical” in human plasma PeptideAtlas was 96% in both batches of samples from the translational study with IQR = 0.9% for the first and 0.8% for the second batch.

**Fig. 1:**
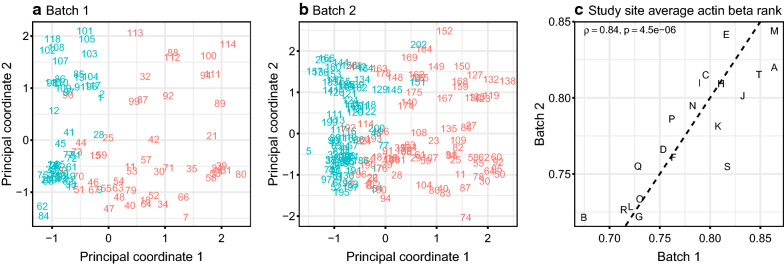
Principal coordinate (classical multi-dimensional scaling) analysis of plasma shotgun proteomics data from two multicenter patient studies **a** and **b** numerical labels represent sample run order, higher values predominantly correspond to positive values of the 2nd principal coordinate; color indicates actin beta levels above (pink) and below (cyan) median; principal coordinates scaled to zero mean and unit variance) and correlation of study site average actin beta rank between two batches (**c**)—letters indicate study sites. In both studies samples with higher levels of intracellular proteins show greater between-sample variability compared to those with lower levels of intracellular proteins

Principal coordinates analysis of shotgun proteomics data revealed two consistent trends across both studies (Fig. 1a, b). Firstly, in both batches, the second principal coordinate—Y axis in Fig. 1a, b)—was closely related to the sample run order. This could be related to performance drift over time of the immunodepletion column or other part of LC–MS/MS proteomics pipeline, which requires the sequential processing of samples. Altered binding capacity of the immunodepletion column or changes in non-specific protein binding may occur over time. Secondly, we noted that the first principal coordinate in both batches was significantly associated with the levels of nominally intracellular proteins representing highly overlapping set between two batches (Additional file 1: Tables S2 and S3). As illustrated in the Additional file 1: Table S4, in total, in the first and second batch from the translational study 76 and 69 proteins respectively pass BH-FDR < 0.05 threshold for their association with the first principal coordinate.

We did not observe a statistically significant correlation between the first principal coordinate and patient demographic features in these two sets (Additional file 1: Table S4). However, we noted that samples collected at particular sites tended to have higher levels of intracellular proteins compared to other sites in both groups, suggesting that the association of the intracellular proteins with the first principal coordinate noted above might be attributable to the differences among translational study sites. This trend showed a highly statistically significant positive correlation across the two studies (Spearman rank correlation = 0.84, p < 10^–5^, Fig. 1c). While intracellular proteins may be found in plasma as a result of tissue leakage or aberrant secretions [21], it appears that variability in sample handling and processing at particular sites is the most likely cause of a site-dependent effect. A list of the proteins associated with this site effect is given in Additional file 1: Table S5. Cytoskeletal and cytosolic proteins are particularly prevalent (e.g. ACTB, VCL, FLNA and TLN1 shown in Additional file 1: Tables S2, S3 and S5) as further reflected by the Gene Ontology categories highly enriched by the proteins showing positive correlation of the levels averaged per study site across two batches (Additional file 1: Table S6).

The presence of these intracellular proteins in plasma from specific collection sites suggests they are released from blood cells (e.g. neutrophils, platelets) during sample processing. Samples with higher levels of these cytoskeletal proteins tend to show a greater degree of dissimilarity in terms of their proteomic profiles in the two sets of samples from our translational study as compared to those with lower levels of them (Additional file 1: Figure S1). In order to further investigate the blood processing factors that could be contributing to the site-effect we have performed several experiments using plasma samples from healthy volunteers.

### Examination of pre-analytical variables associated with proteome variability

**Fig. 2:**
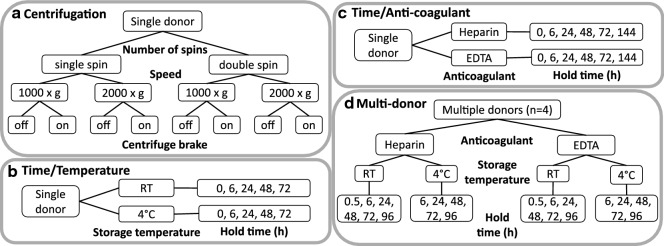
Designs of single donor pilot studies of centrifugation (**a**), hold time and temperature (**b**) and hold time and anti-coagulant (**c**) and multi-donor blood processing experiment (**d**). For multi-donor study a single replicate from each donor was assayed for every combination of processing conditions

After examination of the literature, we identified several preanalytical blood processing variables which might contribute to an increase in intracellular proteins in plasma. These included centrifugation conditions, time between collection and first centrifugation, blood storage temperature prior to processing and type of anti-coagulant. Healthy donor blood was used to test the above variables as summarized in Fig. 2. Improper centrifugation may result in residual blood cells in plasma and lysis of these cells could result in intracellular protein release into the soluble space. Delays in processing blood may also result in protein leakage from blood cells, while storage temperature may influence cell lysis. Likewise, the choice of anticoagulant may introduce some variability. Standardized blood collection protocols generally call for EDTA collection tubes, since EDTA is considered to be more inert than other anticoagulants such as heparin [22]. Since heparin tubes were used for patient samples in our initial studies we compared EDTA and heparin collection tubes here. To further our understanding of the relative impacts of these four factors (centrifugation conditions, sample hold time, temperature and anticoagulant type) and to prioritize them for evaluation in a larger multi-donor experiment we first performed three small-scale single-donor pilot studies as detailed in Fig. 2a–c.

### Proteomic effects of variable centrifugation conditions

In all eight different centrifugation protocols were carried out on blood from a single donor as outlined in Fig. 2a. The number of spins, speed of spin and application of the centrifuge brake were examined. Shotgun proteomics on immunodepleted plasma was carried out and results were assessed for the association between protein levels and these experimental factors. None of the proteins detected (ranging from 267 to 293 proteins per sample, 96% to 98% of proteins annotated as “canonical” in human plasma PeptideAtlas) were found to reach statistical significance upon correction for multiple tests (BH-FDR < 0.05) for the association with the differences in centrifugation protocols and majority of the changes were smaller than twofold (Additional file 1: Figure S2). Ten of the 33 site effect proteins (Additional file 1: Table S5) were detected in this study and none of these proteins showed fold changes greater than 2 between the conditions tested (Additional file 1: Figure S2). The data imply that release of intracellular proteins into plasma during blood processing is not sensitive to variable centrifugation conditions. Additionally, no visible red blood cell hemolysis was observed in any of the conditions tested, suggesting that red blood cell hemolysis was not induced by altered centrifugation. For subsequent experiments blood was spun twice at 1500×*g* with the centrifuge brake applied for both spins.

### Proteomic effects of processing delay and storage temperature

Blood samples from a single donor were processed after storage at room temperature and 4 °C for five different time intervals prior to the first centrifugation (0, 6, 24, 48 and 72 h) as outlined in the Fig. 2b. The resulting ten LC–MS/MS shotgun proteomics profiles of immunodepleted plasma (with 279 to 322 proteins detected per sample, 97% to 98% of which were determined to be “canonical” by human plasma build of PeptideAtlas) were evaluated for the effects of temperature and hold time on the measured protein abundances as further detailed in Supplementary Material (Additional file 1: Section S3.2). Association between storage time and measured protein levels resulted in the largest number of statistically significant differences (51 proteins pass BH-FDR < 0.05 cutoff) most of which correspond to the increase in average protein level with time. Approximately 1/3 of the proteins (14 out of 33) showing correlation with the study site in multi-center translational study (Additional file 1: Table S5) have been detected in this experiment, with 8 of them also passing the statistical significance cutoff (BH-FDR < 0.05) for the effect of storage time corresponding to the increase in their measured levels with time (Additional file 1: Figure S3). As further discussed in Supplementary Material (Additional file 1: Section S3.2), this effect could be at least partially alleviated by storage at 4 °C as compared to room temperature. Two proteins pass BH-FDR < 0.05 cutoff for the main effect of the temperature when analyzed by the regression model with main effects of processing delay, temperature and interaction between them. Further discussion of the impact of model formulation on the significance estimates is provided in Supplementary Material (Additional file 1: Section S3.2). Regardless of the statistical significance, about 10% of proteins demonstrated greater than twofold difference on average between samples stored at room temperature and 4 °C.

### Proteomic effects of processing delay and anticoagulant type

Blood samples from a single healthy donor were collected in EDTA and heparin tubes and stored at room temperature for 0, 6, 24, 48, 72 and 144 h (Fig. 2c). Twelve resulting profiles of protein abundances (with 291 to 343 protein identifications per sample and 94% to 96% of proteins falling in the “canonical” category of PeptideAtlas human plasma build) as measured by LC–MS/MS shotgun proteomics in immunodepleted plasma have been analyzed as further detailed in Supplementary Material (Additional file 1: Section S3.3) for the association of protein levels with anticoagulant type and time. As in the pilot study of time and temperature, the length of processing delay had significant impact on the largest number of proteins (36 proteins pass BH-FDR < 0.05 threshold) most of which increase with time. Of the three pilot experiments conducted on the single donor samples (centrifugation, time and temperature, time and anticoagulant—Fig. 2a–c) this study yielded the highest fraction of proteins—21 out of 34—that also showed positive correlation of their abundances between study sites in the two batches of samples in the translational study. As was the case for the pilot study of time and temperature, the majority of these proteins increased with longer processing delays. Irrespective of the statistical significance, 11% of proteins detected had at least a twofold difference in EDTA versus heparin tubes. Several of them (TLN1, TAGLN2 and TPM4) were significantly correlated across study sites in the translational study. The impact of the power of this pilot study and regression model setup are further described in Supplementary Material (Additional file 1: Section S3.3).

### Proteomic effects of processing delay, storage temperature and anticoagulant

**Fig. 3:**
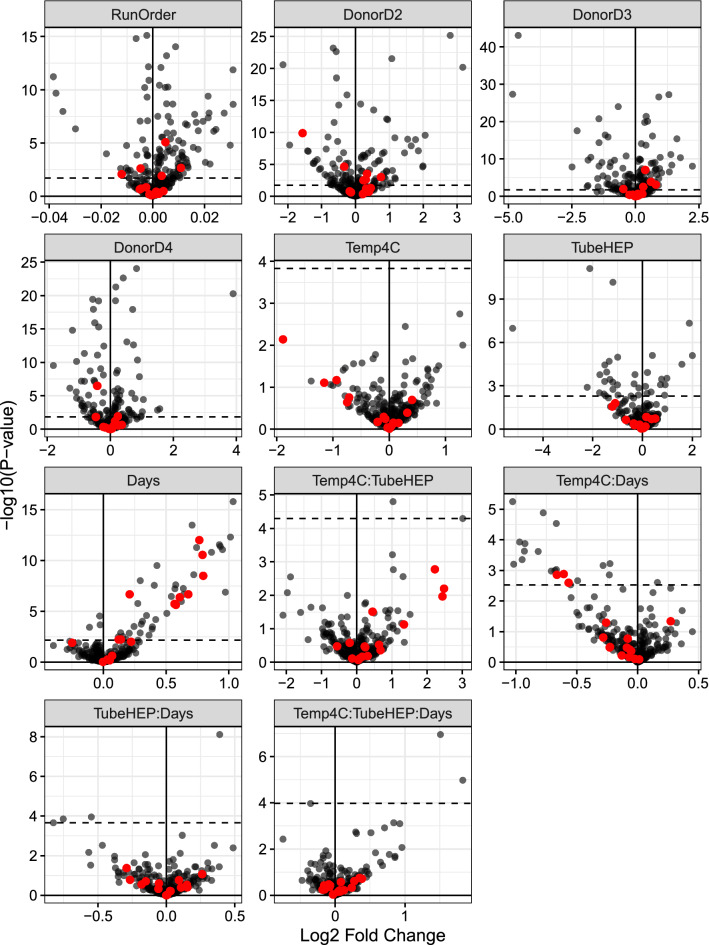
Volcano plots of the effects evaluated in the blood processing study. Horizontal dashes correspond to BH-FDR cutoff of 0.05. Red color indicates proteins with statistically significant (BH-FDR < 0.05) correlation of their abundances across study sites in two batches of samples from the translational study

We carried out an additional experiment with four healthy volunteers in which we varied three factors that showed most pronounced effect on protein abundance in the pilot studies described above (time until first centrifugation, blood storage temperature and anticoagulant) with the goal of assessing their impact across a larger set of samples reflective of between-subject variability (Fig. 2d). As before, shotgun proteomics was carried out on immunodepleted plasma without sample pooling. Samples were randomly ordered prior to immunodepletion and that order was preserved all the way through to LC–MS/MS analysis. Blood was held at room temperature (RT) or 4 °C for 0.5, 6, 24, 48, 72 or 96 h. Both EDTA and heparin blood collection tubes were used. The median count of proteins detected across the resulting 88 samples was 302 (IQR = 14). The median percentage of “canonical” proteins per human plasma build of PeptideAtlas was 93% (IQR = 1.1%). Statistical analysis was carried out on proteomics data to examine main effects and interactions associated with the variables tested. Figure 3 presents results of differential expression analysis in the form of volcano plots. These plots represent statistical significance of each of the factors in the multiple linear regression model (as negative log base 10 of p-value for that factor in the model—vertical axes in plots) versus estimated change in log base 2 protein levels for one unit change in the corresponding factor (horizontal axes in the plots). Distributions of p-values associated with each term in linear models fit to the protein abundance data are provided in the Additional file 1: Figure S5.

As expected, many proteomic differences are driven by inter-individual variability as well as by sample processing order during sample preparation and obtaining LC–MS/MS measurements. Nearly 100 or more proteins were significantly affected by each of these factors (BH-FDR < 0.05). Of the remaining blood processing variables that were tested in this study time until first centrifugation and anticoagulant type (main effects for both) had the most significant impacts on protein changes across all individuals (50 and 36 proteins respectively with BH-FDR < 0.05). Proteins undergoing time-dependent increases included many intracellular proteins (e.g. PFN1, CFL1, GPI, YWHAB—Table 1). Comparison of the rate of change of these proteins’ levels with time to their variability across translational study sites is further described in the Additional file 1: Section S4.3 and Figure S6. Results of this assessment indicated that the scale of the study site variance component is roughly on par with a one day delay in processing time and positively correlated with the rate of change with time for the proteins significantly changing with time in the multi-donor study.

**Table 1:** Top ten proteins with the most statistically significant associations (the lowest BH-FDR adjusted p-values) for the main effect of hold time in multi-donor blood processing study

Accession	Description	logFC	AveExpr	BH-FDR
P02788	LTF: lactotransferrin	1.03	7.29	< 10^–9^
P23528	CFL1: cofilin-1	0.71	6.59	< 10^–9^
P52209-2	PGD: isoform 2 of 6-phosphogluconate dehydrogenase, decarboxylating	1.01	6.31	< 10^–9^
P07737	PFN1: profilin-1	0.76	7.73	< 10^–9^
P06744	GPI: glucose-6-phosphate isomerase	0.92	6.29	< 10^–9^
P30740	SERPINB1: leukocyte elastase inhibitor	0.93	6.16	< 10^–9^
P29401	TKT: transketolase	0.74	7.33	< 10^–9^
P00558-2	PGK1: isoform 2 of phosphoglycerate kinase 1	0.95	6.66	< 10^–9^
P04083	ANXA1: annexin A1	0.86	6.31	< 10^–9^
P31946-2	YWHAB: isoform short of 14-3-3 protein beta/alpha	0.79	6.40	< 10^–9^

Column “logFC” represents estimated fold change on log base 2 scale corresponding to one day delay in processing. Positive sign of “logFC” indicates increase in their levels with time. Column “AveExpr” represents average expression level of each protein on the scale of log base 2 CPM (as further explained in Additional file 1: Section S1)

As a main effect storage temperature did not have a significant proteomic effect. However, temperature and time appear to show an interaction effect, suggesting that higher temperatures may exacerbate the time-dependent release of proteins into plasma. Table 2 shows the top ten proteins most significantly impacted by the interaction between processing delay and temperature, several of which are also shown in Table 1 (main effect of time). The negative sign for the effects of interaction between time and temperature (4 °C as compared to RT) for the proteins shown in Table 2 suggests that the rate of change in their levels is decreased at 4 °C as compared to room temperature.

**Table 2:** Top ten proteins with the most statistically significant associations (the lowest BH-FDR adjusted p-values) for the interaction between temperature and hold time in multi-donor blood processing study

Accession	Description	logFC	AveExpr	BH-FDR
P02788	LTF: lactotransferrin	− 1.03	7.29	0.0019
O14511-2	NRG2: Isoform 2 Of Pro-Neuregulin-2, membrane-bound isoform	− 0.78	7.36	0.0022
P23528	CFL1: cofilin-1	− 0.67	6.59	0.0033
P52209-2	PGD: isoform 2 of 6-phosphogluconate dehydrogenase, decarboxylating	− 0.97	6.31	0.0090
P06744	GPI: glucose-6-phosphate isomerase	− 0.93	6.29	0.0090
P30740	SERPINB1: leukocyte elastase inhibitor	− 0.93	6.16	0.0115
P04083	ANXA1: annexin A1	− 0.82	6.31	0.0115
P00558-2	PGK1: isoform 2 of phosphoglycerate kinase 1	− 0.95	6.66	0.0187
P02655	APOC2: apolipoprotein C-II	− 0.23	9.87	0.0211
P02730	SLC4A1: band 3 anion transport protein	− 1.02	6.15	0.0211

Column “logFC” represents estimated fold change on log base 2 scale due to one day delay in processing at 4C, as compared to room temperature (shown in Table 1). Negative sign for “logFC” indicates lower rate of change with time at lower temperature. Column “AveExpr” represents average expression level of each protein on the scale of log base 2 CPM (as further explained in Additional file 1: Section S1)

The choice of anticoagulant also appears to have an impact on plasma proteome profiles. Here we compared EDTA and heparin tubes and found 36 proteins (BH-FDR < 0.05) to be significantly affected by anticoagulant type (main effect). Table 3 shows the top 10 proteins most significantly affected by the difference between the two types of anticoagulant. The majority of these proteins are detected at lower levels in heparin tube samples (negative log base twofold change for proteins above BH-FDR = 0.05 cutoff in Fig. 3, panel “TubeHEP”). We also monitored the impact of pre-analytical variables on red blood cell hemolysis. Although some known markers of hemolysis (HBA1, PRDX2) show a statistical association with time till first centrifugation, the absolute levels of these proteins are far below what we have previously observed in samples with visible red blood cell hemolysis.

**Table 3:** Top ten proteins with the most statistically significant associations (the lowest BH-FDR adjusted p-values) for the main effect of the anticoagulant type in multi-donor blood processing study

Accession	Description	logFC	AveExpr	BH-FDR
P27169	PON1: serum paraoxonase/arylesterase 1	− 2.12	11.09	< 10^–5^
P00915	CA1: carbonic anhydrase 1	− 1.19	10.14	< 10^–5^
Q99969	RARRES2: retinoic acid receptor responder protein 2	1.88	7.31	< 10^–5^
P80108	GPLD1: phosphatidylinositol-glycan-specific phospholipase D	− 5.22	8.62	< 10^–5^
P12259	F5: coagulation factor V	0.39	11.62	0.00046
P02788	LTF: lactotransferrin	2.00	7.29	0.00046
P02747	C1QC: complement C1q subcomponent subunit C	− 0.99	9.83	0.00051
P00918	CA2: carbonic anhydrase 2	− 1.34	8.29	0.00085
P10124	SRGN: serglycin	1.58	6.99	0.00122
P02746	C1QB: Complement C1q subcomponent subunit B	− 1.51	10.59	0.00122

Negative values represent a decrease (on log base 2 scale of fold change) in protein levels (at zero hours, at room temperature) in heparin tubes compared to EDTA tubes. Column “AveExpr” represents average expression level of each protein on the scale of log base 2 CPM (as further explained in Additional file 1: Section S1)

### Assessment of site-effect protein expression in healthy donor experiment

**Fig. 4:**
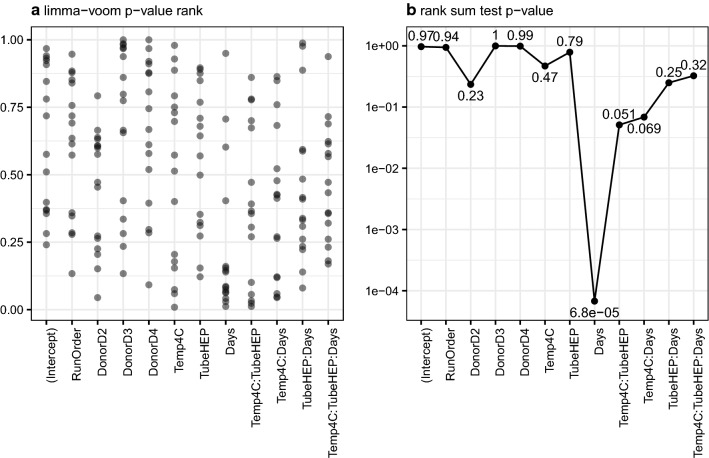
16 proteins with statistically significant (BH-FDR < 0.05) Spearman correlation of study site effect in the two sets of samples from the translational study are predominantly those that achieve higher statistical significance for the main effect of time in the designed study of blood processing factors; additionally, they also, to a smaller degree, tend to demonstrate greater significance for the effects of interaction between time and temperature and temperature and heparin/EDTA type of anticoagulant. Left panel represents ranks of p-values for those 16 proteins across p-values of all proteins for the effect of corresponding study factor—values close to zero correspond to smaller, more significant, p-values; right panel summarizes each scatter on the left as one-sided Wilcoxon p-value representing probability of obtaining sum of ranks lower than observed value by chance

We next asked if the site-effect proteins observed in patient studies were significantly associated with the blood processing variables studied in our healthy multi-donor experiment. In particular, we wanted to determine if one or more of the processing variables examined leads to increases in the number of significant associations for this set of proteins. Figure 4a shows the ranked p-values associated with each variable (or interactions between them) for the 16 site effect proteins (proteins with BH-FDR < 0.05 for Spearman rank correlation with site effect in two groups of samples from the translational study) that were also detected in multi-donor study. From visual inspection of this plot (Fig. 4a) it is immediately apparent that as a group site-effect proteins tend to be the most significantly affected by the processing delay (Days). The majority of these site-effect proteins were significantly impacted by time until first centrifugation (red dots above horizontal dashes representing BH-FDR = 0.05 cutoff in Fig. 3, panel “Days”). The positive sign of corresponding log twofold changes for them suggest that their levels increase with longer processing delays. The results of a one-sided rank sum test comparing p-values for site-effect proteins to all other proteins for each of the effects tested in the multi-donor study are shown in Fig. 4b. By far the most significant (the smallest) p-value shown in this plot is for the effect of hold time (Days), confirming that this variable (processing delay as main effect) has the largest impact on site effect proteins among those tested. Time and temperature (interaction effect) and anticoagulant and temperature (interaction effect) also tend to influence the expression of site-effect proteins, but to a lesser degree. It is interesting to note that storage temperature by itself does not have a significant effect on intracellular protein release—e.g. none of the site effect proteins cross BH-FDR < 0.05 threshold (Fig. 3, panel “Temp4C”). However, intracellular protein release can be exacerbated by temperature as a function of time or anticoagulant. In our two patient sets the same anticoagulant was used for all blood samples (sodium heparin). Therefore, it appears likely that the observation of increased intracellular proteins at particular collection sites can be explained primarily by variable time until first centrifugation with blood storage temperature potentially playing a lesser but still significant role.

## Discussion

Preanalytical variables in blood processing are important factors to consider in interpreting protein-centric plasma measurements. Since biomarker discovery requires large patient groups in order to obtain statistically meaningful results, samples are often acquired from multiple sources (biobanks, hospitals, clinics, contract research organizations, etc.). Procedures and practices relating to handling and processing of blood samples may vary considerably across collection sites. It is unclear how variability across sites may influence proteomics data or whether it could lead to confounding results. Furthermore, few studies have examined this problem in the context of real-world patient samples. Here we have carried out LC–MS/MS shotgun proteomic analysis of plasma from two patient batches (120 and 204 samples, respectively). Samples were drawn from 30 different sites (20 of which contributed samples to both batches). During the analysis of these sets of samples we noted an association between site of collection and elevated intracellular proteins. We reasoned that such an association might relate to handling and processing of blood specimens at particular sites. Although this phenomenon does not inherently prevent one from reaching reproducible conclusions based on proteomics data, its impact could be more pronounced when study factors of interest become inadvertently confounded with variability of the protein levels across study sites. Therefore, we undertook controlled experiments using healthy volunteer blood and examined centrifugation conditions, time before first centrifugation, blood storage temperature and anticoagulant.

The finding that time prior to first centrifugation and storage temperature has the most pronounced impact on intracellular protein levels in plasma is in broad agreement with a previous study. Hassis et al. reported that 41 proteins change in expression after blood was held for 96 h at room temperature [2]. We detected 30 out of these 41 proteins, with 77% being significantly affected by time before first centrifugation (BH-FDR < 0.05, Additional file 1: Figure S7a). Only one protein was found by Hassis et al. as changed significantly after blood was held for 6 h, agreeing with our general observation that longer delays till first centrifugation result in more proteomic variability. Hassis et al. also reported that holding blood at 37 °C for 96 h resulted in changes to 83 proteins. We detected 64 out of these 83 proteins, with approximately 50% being significantly affected by time before first centrifugation (BH-FDR < 0.05, Additional file 1: Figure S7b). In our study we held samples at room temperature and at 4 °C, but not at 37 °C, which may account for the lower concordance in this set of 83 proteins. Nonetheless, both studies demonstrate that blood storage temperature as well as time before first centrifugation are important contributors to plasma proteome variability. Furthermore, our study agrees with the finding of Hassis et al. that single-spun plasma and double-spun plasma do not differ significantly. We have also shown that speed of centrifugation (1000×*g* versus 2000×*g*) and application of the centrifuge brake are not sources of preanalytical variability during plasma proteomic analysis. Additionally, we have detected an association between abundances of multiple proteins and the differences between EDTA and heparin tubes. These proteins were predominantly lower in heparin tubes raising the possibility of heparin interference with quantification of protein abundances by spectral counting methods.

Other studies have shown fewer proteomic differences as a result of increased time before first centrifugation. Zimmermann and colleagues held blood for up to 168 h at room temperature and saw few significant protein changes [4]. However, blood samples were pooled together from 10 patients which precludes from accounting for between patient variability as part of data analysis and may mask small changes in protein abundances in individual patient samples. Furthermore, high abundant proteins were not depleted prior to LC–MS/MS analysis, further limiting detection of intracellular protein release. Kaisar et al. held blood from five healthy volunteers for up to 48 h at ambient temperature [9]. Although samples were pooled together for analysis, depletion of high abundant proteins was carried out. A small number of intracellular proteins were found to be enriched after 48 h including ANXA1, PF4, PFN1, S100A8, S100A9 and THBS1 all of which show significant time-dependent increase in our study (Additional file 1: Figure S7c). Another study collected 100 blood samples from seven different biobanking facilities and held blood for up to 24 h, without seeing any significant proteomic effects [8]. This relatively short timepoint coupled with sample pooling may explain why no changes were observed. In our study we have analyzed individual patient samples (non-pooled) both in our patient studies (324 samples) and healthy volunteer studies (96 samples in multi-donor study). We have held blood at multiple time points up to and including 96 h and we carried out immunodepletion of high abundant proteins on all samples. Therefore, the experimental design used here may be better suited to investigate the effects of preanalytical variables on plasma proteomic results.

We have shown that intracellular protein release occurs when plasma remains in contact with blood cells for prolonged periods. Certain dynamic processes may be driving this phenomenon. Osmolality, defined as the total number of solute particles per kilogram of solvent, is more unstable in unspun blood samples at room temperature relative to previously isolated plasma [23]. In particular this difference is observed within the first three days after isolation. Holding unspun blood at 4 °C partially blocks the increase in osmolality. Water evaporation or glucose consumption by erythrocytes leading to increased lactate production may explain increased molality. Uptake of water by erythrocytes may also cause certain blood analytes to become more concentrated. Oddoze and colleagues observed increased potassium, inorganic phosphate and lactate when blood was held for 24 h [6]. Boyanton et al. found that the concentrations of both albumin and total protein increase significantly after just 24 h, with further increases observed up to 56 h [24]. Therefore, previous findings suggest that allowing plasma to remain in contact with blood cells for extended period of time results in significant biological changes which can affect the outcome of clinical tests. Further work will be required to determine which blood cell types contribute to intracellular protein release. The majority of the proteins which increased with time in our study are expressed at high levels in leukocytes, thrombocytes and erythrocytes [25]. Therefore, it is challenging to determine the precise origin of these proteins.

Our findings underline the need for strict adherence to standard procedures for blood collection and processing, especially when multi-center studies are involved. In 2009 the Early Detection Research Network (EDRN), an initiative of the National Cancer Institute (NCI), published guidelines for plasma and serum collection and processing [22]. The report recommended the use of EDTA tubes and that blood should be processed immediately or held no more than 4 h at 4 °C prior to processing. In instances where deviations from the protocol may occur it becomes important to document those deviations so they can be accounted for in the subsequent data analysis. It is important to note that factors contributing to protein variability across large collections of patient plasma samples may not necessarily prevent meaningful and reproducible conclusions from proteomic data from these samples. This is especially true when study factors of primary interest are uncorrelated with the factors contributing to preanalytical variability. Therefore, it may be helpful to pay close attention to the levels of intracellular proteins in plasma proteomics data to discern the source, be it biological or artefactual, and to evaluate the extent of its confounding with the factors that are of primary interest for the investigators. Furthermore, it would be useful to evaluate the extent of variability of intracellular proteins across samples in the study and correlate them with any documented deviations in sample processing. Intracellular proteins described in this study (e.g. ACTB) may represent practical markers of sample processing bias and therefore need to be evaluated for further development.

## Conclusions

In conclusion, we have demonstrated that variable blood processing procedures may contribute significantly to plasma proteomic variation. When blood is held for prolonged periods prior to processing, intracellular proteins are released into the plasma. Storage temperature and anticoagulant have a lesser but still substantial impact on proteome variation. Processing variability may be more likely to occur in larger multi-center studies where are large numbers of patient samples are needed to adequately power the study. Our results further underscore the importance of processing any collected blood samples as quickly as possible, especially minimizing the time to first centrifugation of the collected sample. Evaluating intracellular proteins (like actin beta) as potential markers of blood sample processing bias will be helpful in future studies. This will be of importance as there is increasing application of plasma proteomics to understand disease and identify biomarkers, and it is essential to understand potential sources of bias in proteomic data.

## Supplementary Information

**Additional file 1.** Supplementary methods;** Tables S1–S6; Figures S1–S7.**

**Additional file 2: Tables S11–S16.**

**Additional file 3: Tables S7–S10.**

## Data Availability

The datasets supporting the conclusions of this article are included within the article and its additional files.

## Abbreviations

ACTB: Actin beta
ANXA1: Annexin A1
BD: Becton, Dickinson and Company
CFL1: Cofilin 1
DMSO: Dimethyl sulfoxide
EDTA: Ethylenediaminetetraacetic acid
FLNA: Filamin A
FTMS: Fourier transform mass spectrometry
GPI: Glucose-6-phosphate isomerase
HBA1: Hemoglobin subunit alpha 1
HPLC: High-performance liquid chromatography
LC–MS/MS: Liquid chromatography combined with tandem-mass spectrometry
MWCO: Molecular weight cut-off
PF4: Platelet factor 4
PFN1: Profilin 1
PRDX2: Peroxiredoxin 2
PSM: Peptide-to-spectrum match
RSLC: Rapid separation liquid chromatography
S100A8: S100 calcium binding protein A8
S100A9: S100 calcium binding protein A9
TAGLN2: Transgelin 2
THBS1: Thrombospondin 1
TLN1: Talin 1
TPM4: Tropomyosin 4
VCL: Vinculin
YWHAB: Tyrosine 3-monooxygenase/tryptophan 5-monooxygenase activation protein beta
