# Supplementary material for: Variable blood processing procedures contribute to plasma proteomic variability
Source: Clin Proteomics. 2021 Jan 19;18:5. doi: 10.1186/s12014-021-09311-3 (PMC7816468; doi:10.1186/s12014-021-09311-3)
Supplement: Supplementary file 1 — Additional file 1. Supplementary methods; Tables S1–S6; Figures S1–S7. [file 12014_2021_9311_MOESM1_ESM.pdf]

Supplementary material: Variable blood processing procedures contribute to plasma proteomic variability

Patrick Halvey, Victor Farutin, Laura Koppes, Nur Sibel Gunay, Dimitrios A. Pappas, Anthony M. Manning and Ishan Capila

12 January, 2021

Contents

1 Proteomics data pre-processing **2**

2 Indication of study site effect in translational samples **2**

2.1 Additional filtering of proteomics profiles from translational study samples 2

2.2 Associations with principal coordinates 3

2.3 Actin beta (ACTB) and similarities between proteomics profiles 5

2.4 Correlation of protein levels between study sites 5

3 Single donor pilot studies **7**

3.1 Centrifugation 7

3.2 Temperature and time 8

3.3 Anticoagulant and time 10

4 Multi-donor blood sample processing study **11**

4.1 Study design and data pre-processing 11

4.2 Analysis of the differences in protein abundance 11

4.3 Hold time effect and study site variance component 14

4.4 Impact of blood processing study factors on the proteins correlated between study sites . . . 14

5 Comparisons to the results reported in the literature **15**

Session Info **16**

Supplementary References **16**

1 Proteomics data pre-processing

For each shotgun LC-MS/MS proteomics dataset analyzed herein, proteins detected with fewer than 1 PSM on average across all samples in the dataset (as well as those depleted by MARS-14 column) have been excluded from the analyses. The impacts on differential protein levels of the experimental factors involved in the processing of blood plasma samples such as time, temperature, centrifugation, etc. (as well as the association between protein levels in the samples from translational study batches and principal coordinates for that data) have been assessed within limma-voom framework [1–7]. This methodology accounts for the dependency between protein variance and average level of protein expression (similar to the trend commonly observed for RNA-seq fragment count data) and provides efficient means for multiple linear regression modeling of differential expression effects in designed experiments.

Default settings available in limma-voom framework were used for normalization and transformation of the count data prior to estimation of the mean-variance trends and fitting of multiple regression linear models. These amounted to: a) shifting data by half a count to offset zero values, b) normalizing such shifted PSM counts for each protein in the sample to the total count of PSMs for that sample and multiplying the result by 10^6 to standardize their relative abundances to count-per-million (CPM), and c) log base 2 transformation of this ratio. No additional normalization (e.g. quantile-quantile or median sweeping) has been applied to the data. Resulting average levels of the proteins in the tables with the results of differential expression analysis by limma-voom are represented by the column AveExpr that displays dataset average of such log base 2 CPM values (e.g. AveExpr=7 corresponds to a protein represented by $2^7 \times 15000/10^6 \approx 2$ PSMs in a proteomic profile with 15K PSMs in total). Visual evaluation of diagnostic plots for mean-variance trends (data not shown) as estimated by limma-voom for each of the datasets analyzed herein indicated that use of these choices in combination with the rest of default settings in limma-voom (e.g. default span for loess fit) resulted in adequate representation of mean-variance dependency for the purposes of differential expression analyses presented below. For further details, please see limma-voom documentation.

Comparison of the proteins detected in each of the datasets described herein to those observed in the plasma build of PeptideAtlas [8] was performed for a subset of proteins detected in each dataset that correspond to the neXtProt [9] entries with protein evidence level 1 (PE1 – evidence at protein level, omitting for simplicity those that were predicted, inferred from homology or with evidence at transcript level only). PE1 proteins in human plasma build "Plasma Non-Glyco 2017-04 – Mapping 2017-05-12" of PeptideAtlas (http://www.peptideatlas.org/hupo/c-hppp/) that were annotated as "canonical" or "not observed" (3509 and 10499 proteins respectively; proteins in "uncertain" and "redundant" categories were excluded from the analysis to streamline the interpretation) were mapped to proteins detected (passing above criteria on a given dataset in its entirety and with non-zero PSM count in a given sample) in each of the samples. Resulting percentage of "canonical" proteins across the datasets as reported in the main text, therefore, represents fraction of proteins detected in corresponding samples analyzed in this study that have been annotated as reliably identified in human plasma, with remaining proteins falling in "not observed" in human plasma category by PeptideAtlas.

2 Indication of study site effect in translational samples

2.1 Additional filtering of proteomics profiles from translational study samples

Additional sample filtering steps have been applied to the data on plasma samples for the two batches of subjects from the translational patient registry which were shipped from multiple clinical investigational sites and stored for varying durations of time. Likely due to this variability in sample collection and storage procedures resulting compendium of proteomics profiles included several obvious outliers vastly different from the majority of the samples in each of the two datasets. These were removed by the application of the following clean-up steps to the data from translational study samples:

Table S1: Demographic attributes of the subjects in the first and second translational study batches of the proteomics measurements that passed pre-processing filtering on PSM counts and average correlation. (BMI=body mass index, SD=standard deviation)

	N	Age, mean(SD)	Female, N(%)	White, N(%)	BMI, mean(SD)
Batch 1	57	56(15)	47(82)	44(77)	29(6.9)
Batch 2	98	55(12)	80(82)	72(73)	31(7.7)

Table S2: Top ten proteins with the highest statistical significance of their association with the first principal coordinate (scaled to zero mean and unit variance) in the first batch from multi-center translational study.

Accession	Description	logFC	AveExpr	adj.P.Val
Q9Y490	TLN1 : Talin-1	2.34	7.28	5.25e-59
P12814	ACTN1 : Alpha-actinin-1	1.94	6.61	1.02e-43
Q5HY54	FLNA : Filamin-A	2.19	7.35	3.74e-40
O43707	ACTN4 : Alpha-actinin-4	1.49	6.14	2.3e-38
P60709	ACTB : Actin, cytoplasmic 1	0.658	10.7	2.3e-38
P08567	PLEK : Pleckstrin	1.25	6.21	1.5e-37
Q6S8J3	POTEE : POTE ankyrin domain family member E	0.701	9.64	6.07e-35
P37802	TAGLN2 : Transgelin-2	1.67	7	3.58e-34
P07951	TPM2 : Tropomyosin beta chain	1.13	5.76	2.42e-33
Q01518	CAP1 : Adenylyl cyclase-associated protein 1	1.27	5.87	2.35e-32

- omitting 2.5% of the samples with the lowest and same percentage of the samples with the highest total PSM counts in each of the sets
- omitting samples with unusually low correlation to the rest of them (with average Spearman's $\rho < 0.8$) – 11 from the first and 15 from the second set – as likely more affected by irregularities in sample handling and preparation procedures

These pre-processing steps resulted in 405 proteins and 103 samples for the first translational batch and 424 proteins and 177 samples for the second one that were used for the following analyses. The number of clinical study sites that contributed samples to the first set was 22 (the number of samples per site ranged from 1 to 15). For the second set samples were collected at 28 study sites (contributing 1 to 21 samples per site). Out of 30 unique study sites that contributed samples to either of the two translational study batches, 20 study sites contributed samples to both first and second sets. Supplementary Table S1 summarizes additional demographic attributes of translational study subjects in each batch corresponding to the samples included in the analyses after pre-processing filtering steps.

2.2 Associations with principal coordinates

Figures 1 a) and b) in the main text show the results of classical multidimensional scaling (MDS, also known as principal coordinates analysis, as implemented in R by `cmdscale`) of the between samples dissimilarities (as one-complements of Spearman correlations between PSM profiles of the samples) in the two sets of translational samples. Principal coordinates were scaled to zero mean and unit variance. Visual inspection of those plots suggests that the second principal coordinate (Y-axes in the plots, "Principal Coordinate 2") is influenced by the order in which plasma samples were processed by LC-MS/MS shotgun proteomics analytical pipeline. This conclusion is consistent with the results of the significance tests for the Spearman's correlation between run order and second principal coordinate in the first ($p = 2 \times 10^{-11}$) and second ($p = 5 \times 10^{-20}$) batch.

Supplementary Tables S2 and S3 list top ten proteins with the most significant (the lowest) p-values for their association with the first principal coordinate (X-axes in the main text Figure 1 a) and b) plots, labeled

Table S3: Top ten proteins with the highest statistical significance of their association with the first principal coordinate (scaled to zero mean and unit variance) in the second translational batch from multi-center study.

Accession	Description	logFC	AveExpr	adj.P.Val
Q9Y490	TLN1 : Talin-1	2.51	7.72	3.26e-92
P12814	ACTN1 : Alpha-actinin-1	2.01	7.02	7.52e-85
P21333-2	FLNA : Isoform 2 of Filamin-A	2.24	7.5	9.45e-79
Q86UX7-2	FERMT3 : Isoform 2 of Fermitin family homolog 3	1.73	6.48	9.45e-79
O43707	ACTN4 : Alpha-actinin-4	1.62	6.73	3.24e-73
B2RCS5	ACTN2 : Actinin, alpha 2, isoform CRA_b	1.49	6.55	2.23e-67
P60709	ACTB : Actin, cytoplasmic 1	0.569	10.8	1.4e-65
Q6S8J3	POTEE : POTE ankyrin domain family member E	0.647	9.73	3.04e-59
P08567	PLEK : Pleckstrin	1.32	6.34	3.45e-58
Q01518	CAP1 : Adenylyl cyclase-associated protein 1	1.34	6.13	3.87e-56

Table S4: Associations of the first principal coordinate with protein levels and demographic attributes of subjects in the two batches from the translational study.

	N(Proteins)	p(Age)	p(Gender)	p(White)	p(BMI)
Batch 1	76	0.21	0.2	0.16	0.57
Batch 2	69	0.75	0.3	0.75	0.27

as “Principal coordinate 1” therein) – for the first and second set respectively. Statistical significance of the association between protein levels and the first principal coordinate was estimated using limma-voom methodology that accounts for the dependency between average protein abundance and its variance. For the purposes of these analyses the first principal coordinate was treated as a continuous variable (scaled to zero mean and unit variance) and no additional covariates were included in the model. Fields labeled “logFC” in Supplementary Tables S2 and S3 represent changes (on log base 2 scale) in the protein abundance with one unit change in the first principal coordinate (i.e. one standard deviation of principal coordinate values). I.e., the range of log base 2 fold changes of protein levels shown in Supplementary Tables S2 and S3 represent average difference between the samples one unit apart as depicted in the main text Figure 1 a) and b). Field “AveExpr” represents average level of corresponding protein expression as log base 2 of count per million PSMs. Lastly, columns “adj.P.Val” represent Benjamini-Hochberg false discovery rate (BH-FDR) for the corresponding p-values from linear models fit by limma-voom.

Highly significant (very small) BH-FDR values for the proteins most significantly associated with the the first principal coordinate in each batch are not particularly surprising. Top principal coordinates that explain most of the variability in the between samples dissimilarity can be expected to have strong association with protein levels in the samples. However, the overlap between protein identities most significantly associated with the first principal coordinate in both sets is far greater than what is expected to be encountered by chance (permutation $p < 0.001$) and biologically it suggests substantial variability of cytoskeletal (e.g. actin, talin, filamin) protein levels in both batches.

Supplementary Table S4 summarizes associations in each batch between the first principal coordinate and protein levels as well as other demographic attributes. Column “N(Proteins)” represents counts of proteins with BH-FDR<0.05 for the association with the first principal coordinate, columns “p(Age)” and “p(BMI)” represent p-values for Spearman correlation between those attributes and first principal coordinate and columns “p(Gender)” and “p(White)” represent p-values from two sample t-tests for the difference between average of the first principal coordinate between genders and white vs. non-white ethnicity. None of the demographic attributes presented in Supplementary Table S4 display consistently remarkable association with the first principal coordinate in both sets of samples from translational multi-center study.

2.3 Actin beta (ACTB) and similarities between proteomics profiles

Actin beta (ACTB) protein is the most highly expressed protein among the top ten proteins that are most significantly associated with the first principal coordinate in both studies (as shown in Supplementary Tables S2 and S3). For the purposes of the following analyses and discussion, actin beta rank-based levels (as within-sample ranks of corresponding PSM counts) will be used as a surrogate marker for each sample cytoskeletal protein content. For instance, indicating whether ACTB levels are above or below their median levels in each sample set in the main text Figure 1 a) and b) and correlation of average levels of ACTB across study sites that contributed samples to each batch in the main text Figure 1 c). Additionally, as shown in the Supplementary Figure S1, samples with higher levels of actin beta (as proxy for cytoskeleton content) in both batches tend to be less similar to each other while those with lower levels of actin beta tend to demonstrate higher correlation between their protein abundances (as represented by PSM counts).

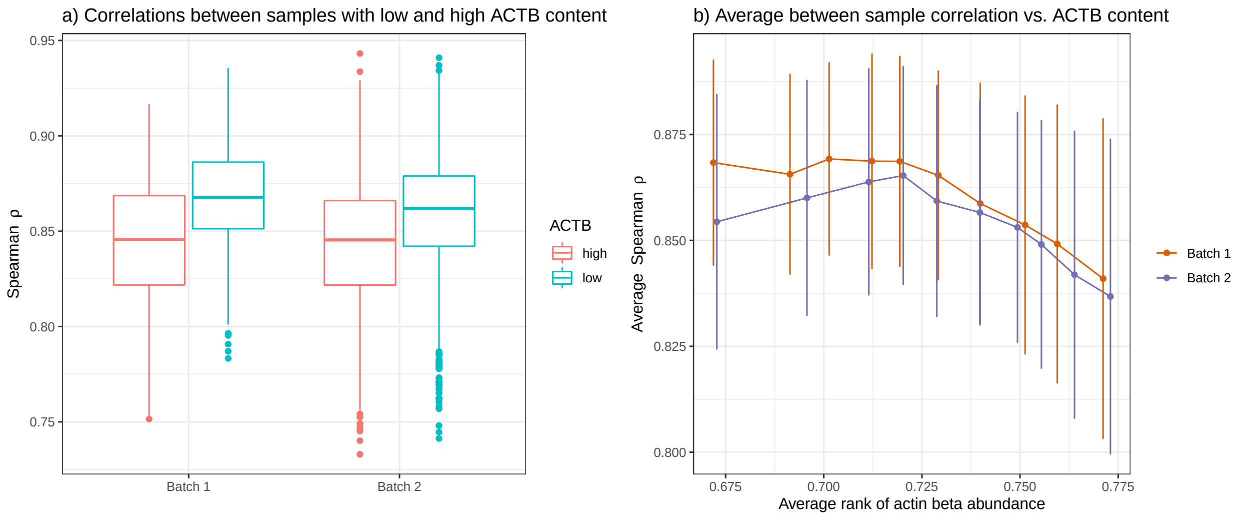

Figure S1: Relationship between actin beta (ACTB) abundance and similarity between samples from two translational studies. a) Non-parametric (Spearman) correlations between protein abundances for the samples with high (above median) and low (below median) ranks of actin beta PSMs. b) Cumulative average correlation among samples ordered by their ACTB content (as rank of corresponding count of PSMs) using their 1st, 2nd, 3rd, etc. deciles as cutoffs. Whiskers indicate standard deviations of corresponding set of correlation coefficients.

2.4 Correlation of protein levels between study sites

Figure 1 c) in the main text shows correlation of average actin beta rank-based levels between study sites that have contributed samples to both translational batches. Similarly, Supplementary Table S5 lists the proteins that have been detected in both sets of samples and passed BH-FDR<0.05 threshold for the significances of Spearman correlations of their study site average rank-based levels between two batches. Relative protein level for a given sample is approximated by within sample rank of corresponding PSM count.

Supplementary Table S6 lists top GO categories (BH-FDR<0.001) enriched for the proteins positively correlated between study sites in two batches. Enrichment of GO categories for positively correlated proteins was estimated using function "geneSetTest" from "limma" package that implements competitive test as described in [10]. Results presented in this table indicate that proteins involved in cell junction, adhesion and cytoskeleton tend to be positively correlated between study sites in these two sets.

Table S5: Top proteins most significantly correlated across study sites in the two translational batches (BH-FDR<0.05)

Accession	Description	Rho	BHFDR
P63104	YWHAZ : 14-3-3 protein zeta/delta	0.95	3.26e-08
P37802	TAGLN2 : Transgelin-2	0.881	2.58e-05
Q01518	CAP1 : Adenylyl cyclase-associated protein 1	0.868	3.69e-05
Q9Y490	TLN1 : Talin-1	0.862	3.69e-05
P67936	TPM4 : Tropomyosin alpha-4 chain	0.862	3.69e-05
P60709	ACTB : Actin, cytoplasmic 1	0.838	0.000116
Q5VU59	TPM3 : Tropomyosin alpha-3 chain	0.832	0.000134
Q13822	ENPP2 : Ectonucleotide pyrophosphatase/phosphodiesterase ...	0.786	0.000766
Q6S8J3	POTEE : POTE ankyrin domain family member E	0.785	0.000766
P08567	PLEK : Pleckstrin	0.767	0.00132
P68032	ACTC1 : Actin, alpha cardiac muscle 1	0.759	0.00141
P12814	ACTN1 : Alpha-actinin-1	0.759	0.00141
P07737	PFN1 : Profilin-1	0.743	0.00221
Q6EMK4	VASN : Vasorin	0.721	0.00396
P00491	PNP : Purine nucleoside phosphorylase	0.717	0.00402
P62261	YWHAE : 14-3-3 protein epsilon	0.708	0.00481
P06733	ENO1 : Alpha-enolase	0.704	0.005
O43707	ACTN4 : Alpha-actinin-4	0.702	0.005
P18206-2	VCL : Isoform 1 of Vinculin	0.699	0.00512
A5A3E0	POTEF : POTE ankyrin domain family member F	0.696	0.00525
P62937	PPIA : Peptidyl-prolyl cis-trans isomerase A	0.693	0.00539
F5GXY9	FCGR2A : Low affinity immunoglobulin gamma Fc region receptor ...	0.69	0.00555
P24593	IGFBP5 : Insulin-like growth factor-binding protein 5	0.678	0.0071
G3V1A4	CFL1 : Cofilin 1 (Non-muscle), isoform CRA_a	0.641	0.0156
Q86UX7-2	FERMT3 : Isoform 2 of Fermitin family homolog 3	0.615	0.0246
Q9BY67-2	CADM1 : Isoform 2 of Cell adhesion molecule 1	0.614	0.0246
Q562R1	ACTBL2 : Beta-actin-like protein 2	0.606	0.027
P31946-2	YWHAB : Isoform Short of 14-3-3 protein beta/alpha	0.605	0.027
Q9H4G4	GLIPR2 : Golgi-associated plant pathogenesis-related ...	0.583	0.0373
Q16610	ECM1 : Extracellular matrix protein 1	0.582	0.0373
P31146	CORO1A : Coronin-1A	0.58	0.0373
O95810	SDPR : Serum deprivation-response protein	0.576	0.039
P61769	B2M : Beta-2-microglobulin	0.559	0.0496

Table S6: Top GO categories (BH-FDR<0.001) enriched for proteins positively correlated across study sites in the two sample sets from translational multi-center investigation. Column Size indicates number of proteins in GO category that have been detected in both batches conditional to the pre-processing steps described above.

GOID	Term	Size	BHFDR
GO:0030054	cell junction	40	1.38e-05
GO:0015629	actin cytoskeleton	17	3.92e-05
GO:0005912	adherens junction	34	3.92e-05
GO:0005924	cell-substrate adherens junction	33	4.64e-05
GO:0005925	focal adhesion	33	4.64e-05
GO:0030055	cell-substrate junction	33	4.64e-05
GO:0070161	anchoring junction	35	4.86e-05
GO:0003779	actin binding	18	5.87e-05
GO:0008092	cytoskeletal protein binding	27	6.89e-05
GO:0003676	nucleic acid binding	29	0.000304
GO:0005829	cytosol	68	0.000435
GO:0045296	cadherin binding	17	0.000527
GO:0050839	cell adhesion molecule binding	37	0.00072
GO:0007015	actin filament organization	17	0.000986

3 Single donor pilot studies

The main goal of the analyses presented below was to select factors involved in the preparation and processing of blood plasma samples that appear to have more profound effect on protein levels in the resulting shotgun proteomics profiles (e.g. higher fraction of more statistically significant effects, larger magnitude of log-fold changes in protein abundances between different levels of study factors) and as such warrant more extensive evaluation. The following three sections summarize those results for each of the three pilot studies conducted.

3.1 Centrifugation

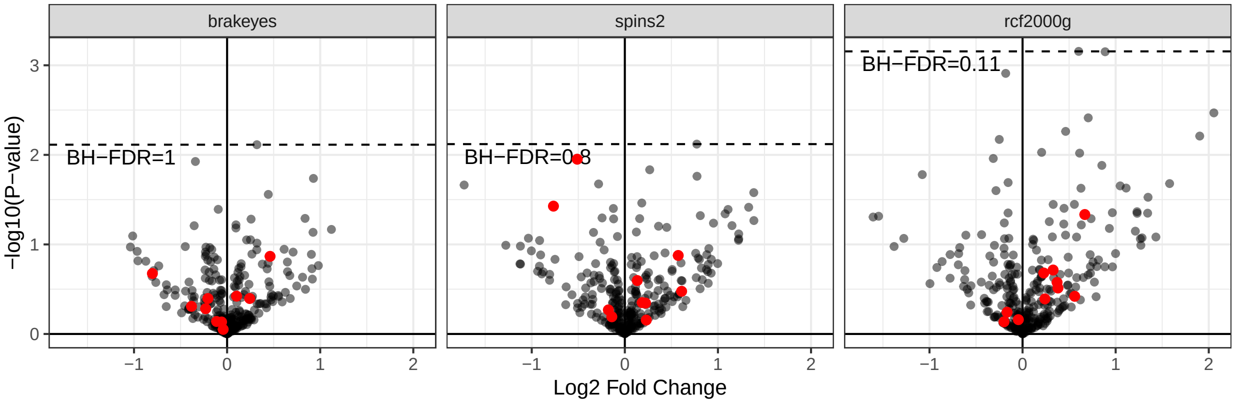

Figure S2: Volcano plots for the effects evaluated in the centrifugation pilot study. Horizontal dashes represent BH-FDR levels as displayed in the plots. Red color indicates proteins showing statistically significant correlation between their study site average abundances in multi-center translational study.

Figure 2a in the main text depicts the study design of a single donor pilot experiment that assessed the

influence of the centrifugation parameters on protein levels as measured by shotgun proteomics in plasma samples. Given very small number of samples assayed for this study – one sample per every combination of each of the three two-level factors analyzed in this pilot study: number of spins (1 or 2), using the brake (yes or no) and acceleration applied to the samples in g-force (or, relative centrifugal force – RCF) units (1000g or 2000g) – linear model that was fit to this dataset using limma-voom framework included only the main effects. Supplementary Figure S2 summarizes in the form of volcano plots the effects of the three factors evaluated in this single donor pilot study investigating the impacts of centrifugation settings on the protein levels in shotgun proteomics profiles of plasma samples. Linear model with three terms representing different levels of these three factors as main effects was fit using limma-voom methodology to the PSM counts of each protein pre-processed as described above (Section 1) that were measured in this study (in total 302 proteins remaining after filtering on the average count of PSMs). Table S7 "Centrifugation" in the supplementary Excel workbook "Additional_File_3.xlsx" includes log fold changes and BH-FDR values for each experimental factor studied herein for every protein analyzed in this study.

Review of the volcano plots depicted in the Supplementary Figure S2 indicates that this pilot study did not detect any remarkable effect of the factors involved in sample centrifugation on the protein abundances in the resulting shotgun proteomics PSM profiles. Once corrected for multiple tests (the number of proteins included in this analysis) no statistically significant effects of braking and number of spins have been found for any of the proteins detected. The lowest values of BH-FDR values for these two effects are close to one that implies that the number of proteins passing those (as well as less conservative) cutoffs on p-values is approximately comparable to what is expected under the null hypothesis of no difference between average protein expression at each level of these two factors. For the effect of acceleration just 3 proteins pass less conservative cutoff of 0.2 on BH-FDR.

Because failure to reach appreciable levels of statistical significance after correction for multiple tests could be also due to the small number of samples resulting in profoundly underpowered study, the magnitudes of the differences in average protein levels across sample processing factors studied in this experiment have been evaluated as well. This assessment resulted in a similar conclusion of paucity (especially as compared to the effects of time, temperature and anticoagulant type presented below) of profound effects of the parameters governing sample centrifugation on the resulting LC-MS/MS shotgun proteomics profiles. For the effects of braking, number of spins and acceleration percentage of proteins showing 2-fold (1 unit difference on log base 2 scale) or greater difference in their abundance in this study was approximately 1%, 5% and 6% respectively, with the largest difference observed across all these three factors of approximately 4-fold (2 units on log base 2 scale).

Lastly, out of 33 proteins shown in Supplementary Table S5 as demonstrating statistically significant correlation of their rank-based abundances averaged per study site between two translational batches, 10 have been also detected in this pilot study (indicated by red color in Supplementary Figure S2). This set of proteins did not display enrichment in the significant associations with any of the factors studied in this experiment (all p-values from two-sample rank sum tests are equal to or greater than 0.1).

3.2 Temperature and time

Two factors evaluated in the second pilot study (see main text Figure 2b for the experiment design) – hold time (0, 6, 24, 48 and 72 hours) and temperature (RT and 4C) – were treated as continuous and categorical respectively for the purposes of linear modeling using limma-voom framework. The multiple regression linear model used to analyze this dataset to access the impact of these two factors on protein levels as measured by LC-MS/MS shotgun proteomics included terms for both the main effects of time and temperature as well as the term for two-way interaction between them. The interaction term allows the model to account for potential difference in the rate of change in protein levels with time at different temperature. The shotgun proteomics data in the form of PSM counts has been pre-processed as described above (Section 1) resulting in 337 proteins that were used to estimate mean-variance trend and to fit linear models estimating effects of temperature and time on their respective abundances. Log fold changes and corresponding BH-FDR significance estimates for every term in the linear model are listed in Table S8 "Temperature x Time" (supplementary Excel workbook "Additional_File_3.xlsx") for each protein included in the analysis.

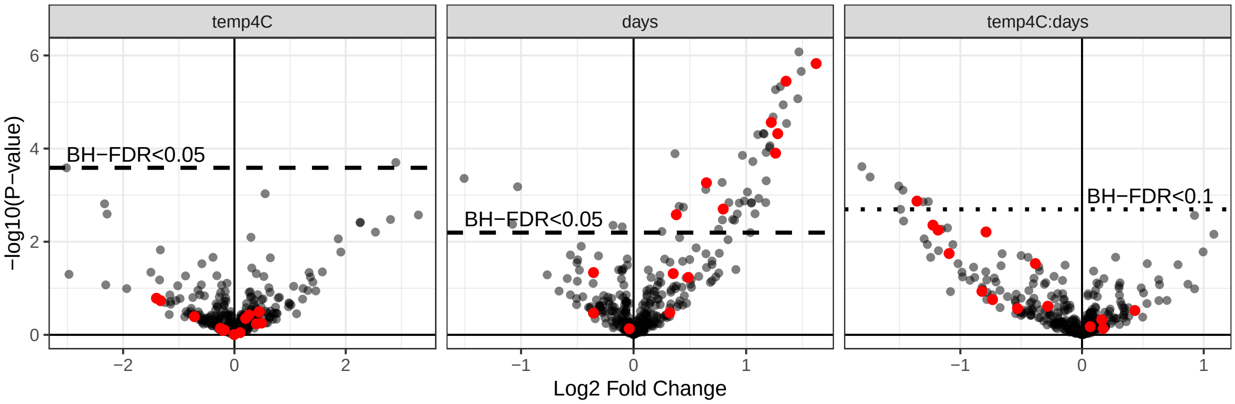

Figure S3: Volcano plots for the effects evaluated in the pilot study of time and temperature. Horizontal dashed and dotted lines represent cutoffs for BH-FDR of 0.05 or 0.1 respectively as shown in the plots. Red color indicates proteins showing statistically significant correlation between their study site average abundances in multi-center translational study.

Volcano plots presented in Supplementary Figure S3 summarize the effects of processing delay and temperature as well as their interaction (difference in rate of change with time at 4C and room temperature) on the levels of the proteins detected in this study. Horizontal dashes indicate thresholds (BH-FDR<0.05, or BH-FDR<0.1 if no proteins pass BH-FDR<0.05 cutoff) on statistical significance of the effects of these factors on each protein after BH-FDR correction for multiple tests (number of proteins detected in this dataset).

It can be readily observed from this figure (panel "days" in Supplementary Figure S3) that time has the most pronounced effect on the protein levels in this study with 51 proteins passing BH-FDR<0.05 cutoff. Majority of these points (above the horizontal dashes in the plot) correspond to positive values of log base 2 fold change that suggests that their levels as measured in this experiment increase for the longer processing delays (somewhere at the rate of $2^{0.5} = 1.4$ to $2^{1.5} = 2.8$ fold per day). Out of 33 proteins showing significant correlation with study site in multi-center translational study (Supplementary Table S5) 14 were detected in this experiment, subject to pre-processing steps outlined above (indicated by red color in Supplementary Figure S3). 8 of them passed BH-FDR<0.05 cutoff and showed positive log base 2 fold change for the effect of time indicative of the increase in processing delay potentially leading to elevated levels of the proteins that also demonstrate correlation of their average abundances between translational study sites.

Additionally, a smaller number of proteins passes less conservative threshold (BH-FDR<0.1; none pass 0.05 cutoff) for the interaction between time and temperature (panel "temp4C:days" in Supplementary Figure S3). All of them have negative values (by their absolute magnitude roughly on par with those observed for the effect of time) suggesting that for some of proteins rate of the increase of their measured abundance with time is lower at 4C than at room temperature. This interpretation is further supported by pronounced negative correlation (Spearman $\rho = -0.64$) between effects of time and interaction between time and temperature across all 337 proteins included in this analysis. This implies that the proteins with the fastest increase in their abundance at room temperature tend to be also those that display largest decrease in this rate at 4C. Proteins showing significant correlation between study sites in the translational study batches (Supplementary Table S5, red color in Supplementary Figure S3) are predominantly those with negative values for log base 2 fold change for the interaction between time and temperature, suggesting that the increase in their levels is slower at 4C than at room temperature.

Lastly, there are only a couple of proteins passing BH-FDR<0.05 cutoff for the main effect of the difference between room temperature and 4C while the majority of the proteins have more than order of magnitude higher (less statistically significant) p-values for this effect. Overall, the fraction of the proteins showing greater than 2-fold difference (irrespective of its statistical significance) between room temperature and 4C after accounting for the effects of time and interaction between time and temperature (absolute value of log

base 2 fold change greater than one for the main effect of temperature) is 11%.

The statistical significance of estimated effects of time and temperature can be impacted by both sample size, and therefore power, of the dataset as well as the setup of the regression models. For this pilot study only one observation was obtained for each combination of the processing delay and temperature levels and the linear model fit to this data included both main effects of temperature and time and the interaction between them, thus enabling evaluation of potential difference between the rates of change in protein expression levels at room temperature and 4C. For this model, two proteins passed BH-FDR<0.05 threshold for the main effect of temperature, i.e. for the estimated average difference between protein levels at these two temperatures when processing delay is equal to zero (to be representative of the samples processed promptly after their collection). Evaluating average differences between protein levels after processing delay of one day at room temperature and 4C for the model including both the main effects and interaction term yields 9 proteins passing BH-FDR<0.05 threshold. Alternatively, modifying the regression model to include only the main effects of temperature and processing delay by omitting the interaction term between them (therefore assuming constant difference between protein levels from the samples held at room temperature and 4C across the entire range of processing delays evaluated herein) yields 26 proteins passing BH-FDR<0.05. Cumulatively, these results suggest increasing with time differences between selected protein levels for the samples held at 4C and room temperature and emphasize importance of these two factors for further evaluation.

3.3 Anticoagulant and time

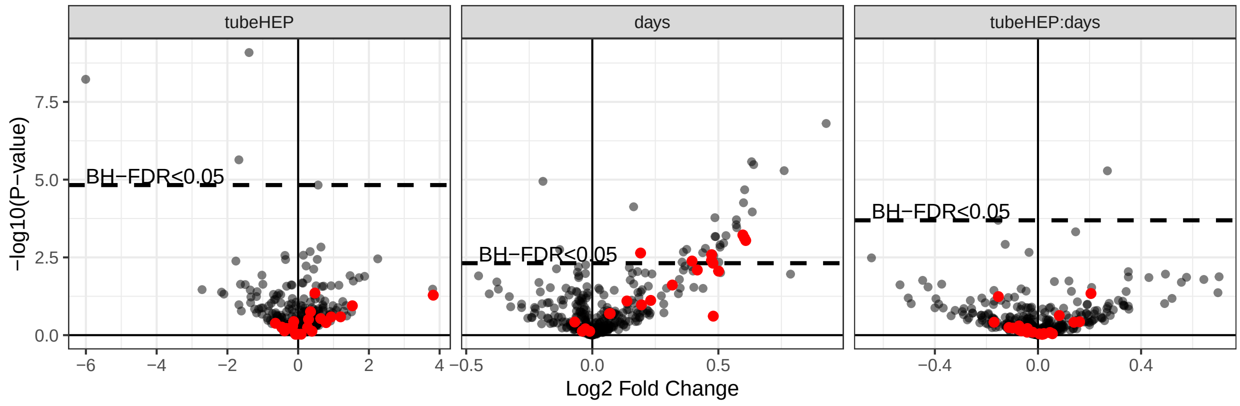

Figure S4: Volcano plots for the effects of anticoagulant type and incubation time evaluated in the pilot study. Horizontal dashes correspond to BH-FDR threshold of 0.05. Red color indicates proteins showing statistically significant correlation between their study site average abundances in multi-center translational study.

Study of the type of anticoagulant tube used for blood collection evaluated two types of anticoagulant – EDTA and heparin – that was treated as a categorical attribute for the purposes of linear modeling. Plasma shotgun proteomics profiles were obtained at 6 timepoints (0, 6, 24, 48, 72 and 144 hours) of incubation in EDTA and heparin tubes. Hold time (in days) was treated as a continuous variable by the linear model. Figure 2c in the main text displays the experiment layout. Additionally, the term for two way interaction between anticoagulant tube and time was added to the model to account for possible differences in the rates of change of protein abundance with time in EDTA and heparin tubes. The pre-processing of PSM counts from the shotgun proteomics profiles for the samples assayed in this study was conducted as described above (Section 1) and resulted in 359 proteins that have been evaluated for the impact of anticoagulant tube type and hold time on their measured levels. For each of these proteins corresponding log fold changes and BH-FDR values for every term in the linear model can be found in supplementary Excel workbook “Additional_File_3.xlsx” (Table S9 “Anticoagulant x Time”).

Results of this analysis are presented in the Supplementary Figure S4 in the form of volcano plots depicting

both statistical significance (as negative log base 10 of p-values – vertical axes) and magnitude of difference (as log base 2 fold change) corresponding to one unit of change in the experimental factor (EDTA or heparin tube or one day of hold time). Horizontal dashes in the plots shown in Supplementary Figure S4 correspond to the cutoff of 0.05 on the corrected for multiple tests (BH-FDR) significance estimates from the linear models for each of those effects (type of the tube, rate of change with time and difference in rates of change between two type of anticoagulant tubes) simultaneously modeling all three of them. Red color indicates 21 proteins that have been detected in this pilot experiment out of 33 proteins that showed statistically significant correlation between study sites in multi-center translational sets (Supplementary Table S5).

Consistently with what has been also observed in the previous analysis (Section 3.2) of the effects of temperature and time, the largest number of proteins – 36 – that pass BH-FDR=0.05 threshold has been obtained for the effect of time (panel "days" in Supplementary Figure S4). Majority of these changes have positive sign (including those that demonstrated correlation with study site in multi-center translational batches). This suggests that these proteins increase their abundance as measured by shotgun proteomics for longer processing delays (at a rate of approximately $2^{0.5} = 1.4$ to $2^1 = 2$ fold per day that is roughly comparable to what was observed in the pilot study of time and temperature described above in Section 3.2).

The counts of proteins passing cutoff of BH-FDR<0.05 for the main effect of anticoagulant tube type (panel "tubeHEP" in Supplementary Figure S4) and interaction between tube type and hold time (panel "tubeHEP:days") are relatively small – 4 for the anticoagulant tube type and 2 for the interaction between tube type and time. Similarly to the observations made in Section 3.2 regarding the interplay between regression model setup and statistical significance of the findings, omitting the interaction term increases the number of proteins passing BH-FDR<0.05 cutoff for the main effect of anticoagulant type to 17 and evaluating average differences between protein levels in these two types of anticoagulant tubes after one day of processing delay results in 9 proteins passing BH-FDR<0.05 threshold for this effect. Regardless of their statistical significance, the percentage of the proteins demonstrating larger than 2-fold difference in their average protein levels between two anticoagulant tubes (absolute value of log base 2 fold change greater than one for the main effect of the anticoagulant in the model including also terms for time and its interaction with anticoagulant type) is 11%. Three of them (TLN1, TAGLN2, TPM4) are also those that showed significant correlation between study sites in multi-center translational study.

4 Multi-donor blood sample processing study

4.1 Study design and data pre-processing

The main text Figure 2d displays design of the study that evaluated effects of the temperature, time and anticoagulant type on plasma protein levels as evaluated by shotgun proteomics across multiple ($n = 4$) donors. Each combination of the three study factors (temperature, time and type of the anticoagulant tube) was applied to the blood samples from the same four donors. Samples were randomized with respect to the sample preparation and processing order in LC-MS/MS shotgun proteomics analytical pipeline. Removal of the low abundance proteins as described in Section 1 above resulted in 337 proteins available for the assessment of the influence of study factors on the differences in their measured levels.

4.2 Analysis of the differences in protein abundance

Linear model employed for the analysis of differences in protein levels as measured in this study accounted for the effects of sample run order, donor identity, temperature (RT or 4C), duration of processing delay (in days, modeled as a continuous covariate) and the type of anticoagulant tube. It also included three interaction terms for all pairwise combinations of temperature, time and type of anticoagulant to assess potential mutual inter-dependency among the effects of each of these three factors, as well as the term for a three-way interaction between hold time, temperature and anticoagulant type. Such three-way interaction term allows the model to account for potential difference in the impact of temperature on the rate of the

change of protein levels with time that (impact of temperature) is also different between EDTA and heparin tubes.

Supplementary Figure S5 summarizes results of differential analysis of protein levels in this multi-donor blood processing study data by limma-voom in the form of histograms of p-values for each of the factors included in the model. This representation is complementary to the Figure 3 in the main text summarizing them in the form of volcano plots. These plots represent statistical significance of each of the factors in the multiple linear regression model (as negative log base 10 of limma-voom p-value for that factor in the model, vertical axes in the main text Figure 3 plots) presented above versus estimated change in log base 2 protein levels for one unit change in the corresponding factor (horizontal axes in the main text Figure 3 plots). Supplementary Excel workbook "Additional_File_3.xlsx" (Table S10 "Multi-donor") exhibits log fold changes and BH-FDR estimates for every protein analyzed here for every term in the statistical model as depicted in the Supplementary Figure S5 and Figure 3 in the main text.

Concordantly with the profound skew towards zero seen in the histograms of limma-voom p-values shown in Supplementary Figure S5, large number of proteins passing BH-FDR<0.05 threshold (above horizontal dashes in the main text Figure 3 plots) for the effects of the run order of the samples ("RunOrder" panel) and donor identity (panels "DonorD2", "DonorD3" and "DonorD4") reflects significant impact of those factors on the average protein levels in the LC-MS/MS shotgun proteomics profiles for immunodepleted blood plasma samples. For the sample processing order this is consistent with the observations made for the samples from the translational batches above.

Of the factors that this experiment was designed to study (time, temperature, anticoagulant), the highest number of proteins passing BH-FDR<0.05 cutoff was observed for the main effect of the duration of processing delay (panel "Days" in Supplementary Figure S5). The top ten proteins achieving the highest statistical significance for the effect of processing delay on their abundance (after accounting for the rest of the factors included in the model) are shown in Table 1 in the main text. The main effects of time on protein abundance levels are predominantly positive (most of the proteins above horizontal dashes in panel "Days" in the main text Figure 3 show positive sign of their log base 2 fold change) suggesting that their levels in shotgun proteomics samples gradually increase for longer durations of processing delay (approximately at the rate between $2^{0.25} = 1.2$ and $2^1 = 2$ fold per day).

The next two largest numbers of proteins passing BH-FDR<0.05 cutoff for the factors studied in this experiment can be seen for the main effect of anticoagulant tube type (panel "TubeHEP", the top ten proteins with the most statistically significant associations are shown in main text Table 2) and for the interaction between storage temperature and processing delay (panel "Temp4C:Days"). This suggests that protein levels detected in LC-MS/MS shotgun proteomic profiles of plasma samples (after accounting for variability due to sample processing order and between subject variability) can vary for some of the proteins between samples stored in EDTA and heparin anticoagulant tubes and that the rates of their change with time can be different at room temperature and 4C. Predominantly negative effect of the two-way interaction between temperature and time (most of the proteins above horizontal dashes in panel "Temp4C:Days" in the main text Figure 3 have negative values of their log base2 fold change) suggests that the rate of their change with time is lower at 4C than at room temperature (concordantly with the observation made for the pilot study of time and temperature above).

For the pairwise interaction terms between temperature and anticoagulant type (panel "Temp4C:TubeHEP") or anticoagulant type and time (panel "TubeHEP:Days") as well as the three-way interaction term between temperature, anticoagulant type and processing delay (panel "Temp4C:TubeHEP:Days") the numbers of proteins passing BH-FDR<0.05 threshold are much smaller (in single digits). This can be interpreted that for the majority of the proteins effects of these factors are unremarkable. Interestingly, by far the highest statistical significance of the three-way interaction term is observed for the lactotransferrin (LTF; P02788, BH-FDR $= 3.7 \times 10^{-5}$) that could be indicative of the difference in the rates of neutrophil degranulation between heparin and EDTA tubes and at 4C and room temperature.

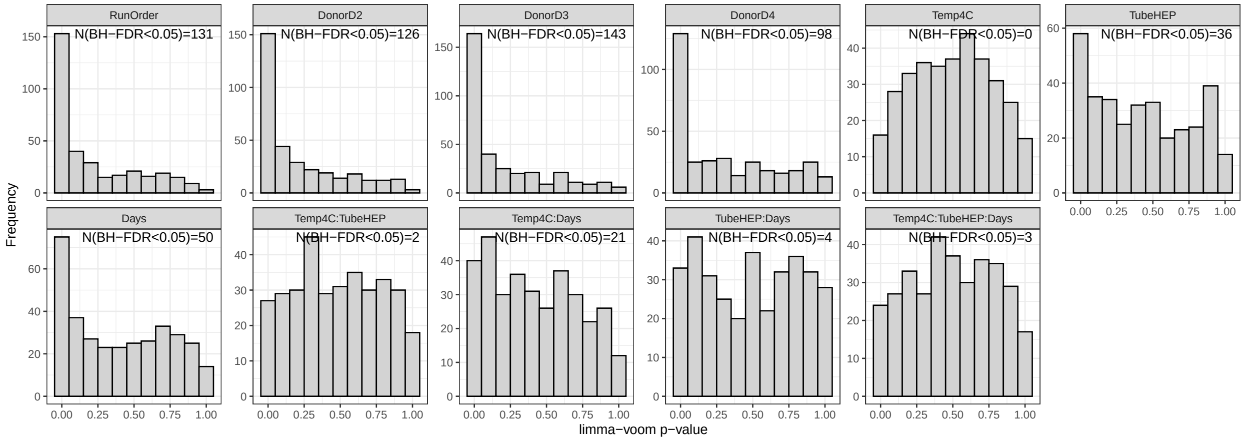

Figure S5: Distributions of p-values for the effects of blood processing variables tested in the multi-donor experiment and interactions between them on the protein abundances in immunodepleted plasma samples as measured by LC-MS/MS shotgun proteomics.

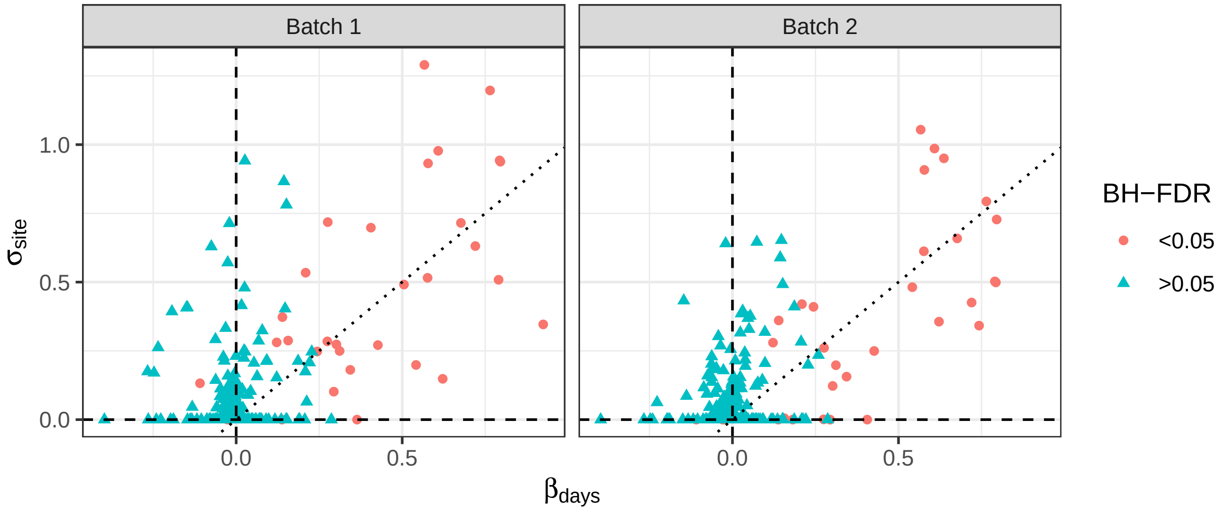

Figure S6: Magnitude of study site variance component (as standard deviation, σ_{site}) in the two batches of translational samples vs. magnitude of the processing delay effect (β_{days}) in multi-donor designed experiment. Diagonal dots represent $y = x$ identity line. Red dots indicate proteins with BH-FDR<0.05 for the effect of the processing delay in multi-donor designed experiment.

4.3 Hold time effect and study site variance component

Supplementary Figure S6 compares the magnitude of the variance components estimated for translational study sites to the estimated rate of change of protein levels with the increase of processing delay in multi-donor designed experiment. Each panel represents all proteins detected both in the corresponding batch of samples from the translational study and multi-donor designed experiment. Variance components were estimated by a random effects model as implemented in R package `lme4` with random effects representing study sites and study subjects that was fit for the estimated abundances (log base 2 transformed, normalized to total count of PSMs per sample, shifted by 1/2) of each protein. Proteins with study site variance components estimated to be close or equal to zero (on $y = 0$ horizontal dashes in the plots) represent proteins with variance attributable to the differences between study sites that is much smaller than that between or within the study subjects. Proteins with BH-FDR<0.05 for the effect of processing delay in multi-donor designed experiments are represented by red dots in the plots.

Magnitude of the study site variance component is represented in these plots as a standard deviation (i.e. square root of variance), σ_{site}, that was calculated on the same (log base 2 fold change in normalized PSMs) scale as the rate of change in protein level, β_{days}, associated with processing delay of one day. Dotted diagonals in the plots represent equality of these two statistics $\sigma_{site} = \beta_{days}$, so that the points falling on this diagonal represent proteins with variability attributable to study site differences equal to the processing delay of one day. Proteins corresponding to the significant (BH-FDR<0.05, red dots) effect of processing delay in multi-donor designed study are approximately evenly scattered around (above and below) $\sigma_{site} = \beta_{days}$ diagonals in both plots, suggesting that for those proteins magnitude of the variability attributable to the differences between study sites is roughly on par with the effect of processing delay by one day.

Additionally, positive correlation between the magnitude of variance components attributed to the differences between study sites and the magnitude of the effect of the processing delay in the multi-donor experiments for the proteins that pass BH-FDR<0.05 threshold is observed for each of the two translational batches (Spearman's $\rho = 0.6$ and 0.72 for the 1st and 2nd batch respectively). Permutation controls randomly scrambling assignments of study subjects to study sites prior to variance components analysis infrequently yield correlation coefficients of this magnitude in each of the two translational batches ($p = 0.008$ for batch 1, $p = 0.002$ for batch 2, both passing statistical significance cutoff of $p < 0.05$). This suggests that proteins demonstrating greater effects of holding time in multi-donor designed study that are also statistically significant additionally tend to be the ones that have larger variance components attributed to the differences between study sites in both batches of translational samples.

4.4 Impact of blood processing study factors on the proteins correlated between study sites

Impact of the factors evaluated in the designed multi-donor blood processing study on the proteins positively correlated between study sites in the samples from the two translational investigation batches has been summarized by a rank sum test. Top 33 proteins with BH-FDR<0.05 (Supplementary Table S5) for the significance of correlation of their rank-based abundances between study sites in two translational investigation batches ($\rho \geq 0.56$) were selected as those that were most influenced by the sample variability across study sites. Of these proteins approximately half (specifically, 16) were also detected in the multi-donor blood processing study and included in the analyses subject to the pre-processing steps described above. Significance estimates in the form of limma-voom p-values for each factor evaluated in the blood processing study have been summarized for this ("study site") set of proteins by a two-sample rank sum (Wilcoxon-Mann-Whitney) test. Resulting one-sided rank sum test p-values testing whether limma-voom p-values in the "study site" set are lower than the p-values for this study factor for the rest of the proteins analyzed in the designed experiment of blood processing factors are presented in the main text Figure 4 b).

Two sample rank sum test of the p-values for each experimental factor in the blood processing study for the "study site" proteins versus the rest of the proteins in the dataset allows for this comparison to account for the non-uniform distribution of limma-voom p-values for a given model term. In other words, result of such

comparison is independent of how significant was the effect of a given model term on the protein expression levels in the designed blood processing study.

For instance, significance estimates for the intercept term in the model – labeled as "(Intercept)" in the main text Figure 4 – are highly skewed towards zero (all $p < 10^{-12}$). This effectively rules out for every protein in the dataset the null hypothesis of zero level of their shifted by half a count and log base 2 transformed PSM count per million at the base levels of all study factors and, unsurprisingly, indicates that their abundances are far higher than half a PSM per million – as every protein in this analysis has been detected by at least 88 PSMs across the entire dataset of 981863 PSMs in total.

By being closely related to the average level of protein expression, limma-voom p-values for the intercept term are also inversely correlated with the average level of protein expression in the blood processing study data (Spearman $\rho = -0.85$ on 337 proteins). For these reasons – mostly indicative of protein abundance, limma-voom p-values near zero and coefficients all positive far from zero – the intercept term, as non-informative, was also omitted from summary of the model fit effects in the form of volcano plots and histograms shown in the main text Figure 3 and Supplementary Figure S5 respectively.

However, the ranks of the p-values for the intercept term of the "study site" proteins do not demonstrate more profound skew towards the lower values as compared to the rest of limma-voom p-values for the intercept (Figure 4 a) in the main text). In fact, corresponding one-sided rank sum test p-value close to one, as shown in the main text Figure 4b), suggests that, if anything, "study site" proteins tend to have higher (relative to the rest of the proteins in blood processing data) limma-voom p-values for the intercept term and, consequently, on average as a set are less abundant than the rest of the proteins in this dataset.

Further discussion regarding the effects of the factors evaluated in the designed multi-donor blood processing study on the proteins showing positive correlations between study sites in multi-center translational study batches can be found in the main text.

5 Comparisons to the results reported in the literature

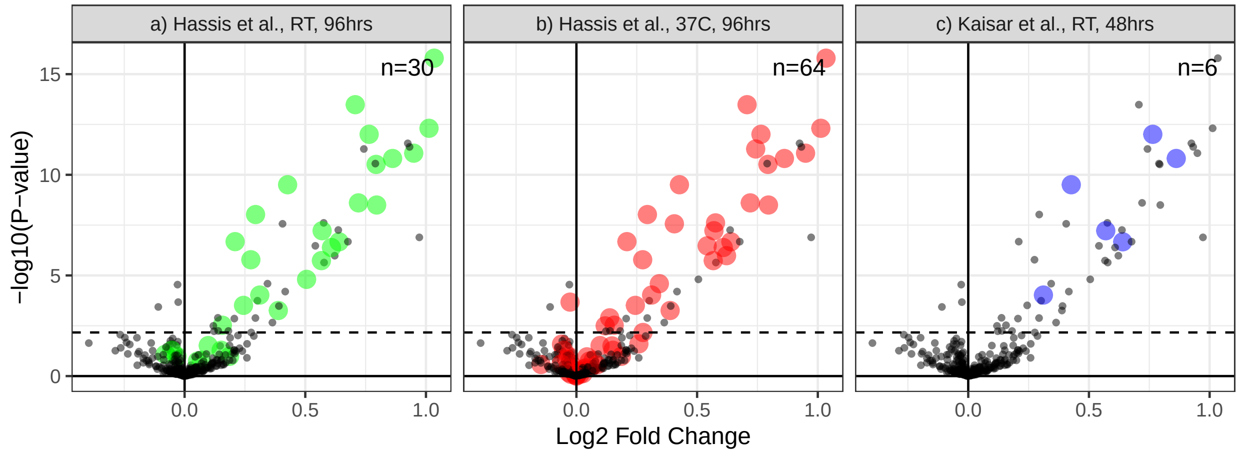

Figure S7: Volcano plots of the effect of processing delay from the multi-donor blood processing study with the proteins reported in the literature indicated by size and color of the symbols. Horizontal dashes correspond to BH-FDR cutoff of 0.05.

Supplementary Figure S7 depicts effect of processing delay observed in multi-donor blood processing study in the form of volcano plots with three sets of proteins reported in the literature (in Hassis et al. [11] after 96 hours at room temperature and 37C and in Kaisar et al. [12] after 48 hours at ambient temperature) indicated

by size and color of the cues. It is readily apparent from visual evaluation of these plots that the majority of the proteins showing significant (BH-FDR<0.05) association with hold time in multi-donor study reported here are also those that demonstrated significant changes in Hassis et al. work (both at room temperature and at 37C) and that abundances of all six proteins shortlisted by Kaisar et al. as enriched after 48 hours at ambient temperature also significantly increase with the processing delay in our multi-donor experiments.

Session Info

The information below represents versions of R / Bioconductor software used to generate computational results presented above. Complete Rmarkdown version of the data analysis procedure reproducing results presented in this manuscript is available upon reasonable request.

- R version 3.6.2 (2019-12-12), `x86_64-w64-mingw32`
- Locale: `LC_COLLATE=English_United States.1252`, `LC_CTYPE=English_United States.1252`, `LC_MONETARY=English_United States.1252`, `LC_NUMERIC=C`, `LC_TIME=English_United States.1252`
- Running under: `Windows 10 x64 (build 17763)`
- Matrix products: default
- Base packages: base, datasets, graphics, grDevices, methods, parallel, stats, stats4, utils
- Other packages: AnnotationDbi 1.48.0, Biobase 2.46.0, BiocGenerics 0.32.0, doParallel 1.0.15, doRNG 1.8.2, foreach 1.4.7, ggplot2 3.3.0, GO.db 3.10.0, gridExtra 2.3, IRanges 2.20.2, iterators 1.0.12, kableExtra 1.1.0, limma 3.42.2, lme4 1.1-21, Matrix 1.2-18, org.Hs.eg.db 3.10.0, plyr 1.8.6, RColorBrewer 1.1-2, reshape2 1.4.3, rngtools 1.5, S4Vectors 0.24.3, SuppDists 1.1-9.5, xlsx 0.6.1
- Loaded via a namespace (and not attached): assertthat 0.2.1, bit 1.1-15.2, bit64 0.9-7, blob 1.2.1, bookdown 0.17, boot 1.3-23, codetools 0.2-16, colorspace 1.4-1, compiler 3.6.2, crayon 1.3.4, DBI 1.1.0, digest 0.6.25, dplyr 0.8.5, evaluate 0.14, farver 2.0.3, glue 1.3.2, grid 3.6.2, gtable 0.3.0, highr 0.8, hms 0.5.3, htmltools 0.4.0, httr 1.4.1, knitr 1.27, labeling 0.3, lattice 0.20-38, lifecycle 0.2.0, magrittr 1.5, MASS 7.3-51.4, memoise 1.1.0, minqa 1.2.4, munsell 0.5.0, nlme 3.1-142, nloptr 1.2.1, pillar 1.4.3, pkgconfig 2.0.3, purrr 0.3.3, R6 2.4.1, Rcpp 1.0.4, readr 1.3.1, rJava 0.9-11, rlang 0.4.5, rmarkdown 2.0, RSQLite 2.2.0, rstudioapi 0.11, rvest 0.3.5, scales 1.1.0, splines 3.6.2, stringi 1.4.6, stringr 1.4.0, tibble 2.1.3, tidyselect 1.0.0, tools 3.6.2, vctrs 0.2.4, viridisLite 0.3.0, webshot 0.5.2, withr 2.1.2, xfun 0.12, xlsxjars 0.6.1, xml2 1.2.5, yaml 2.2.1

Elapsed compilation time: 6056.28 sec.

Supplementary References

1. Law CW, Chen Y, Shi W, Smyth GK. Voom: Precision weights unlock linear model analysis tools for rna-seq read counts. Genome Biology [Internet]. 2014;15:R29. Available from: https://doi.org/10.1186/gb-2014-15-2-r29

2. Ritchie ME, Phipson B, Wu D, Hu Y, Law CW, Shi W, et al. Limma powers differential expression analyses for rna-sequencing and microarray studies. Nucleic Acids Research [Internet]. 2015;43:e47. Available from: http://dx.doi.org/10.1093/nar/gkv007

3. Phipson B, Lee S, Majewski IJ, Alexander WS, Smyth GK. Robust hyperparameter estimation protects against hypervariable genes and improves power to detect differential expression. Ann Appl Stat [Internet]. The Institute of Mathematical Statistics; 2016;10:946–63. Available from: https://doi.org/10.1214/16-AOAS920

4. Schwammle V, Leon IR, Jensen ON. Assessment and improvement of statistical tools for comparative proteomics analysis of sparse data sets with few experimental replicates. Journal of proteome research. ACS Publications; 2013;12:3874–83.

5. D'Angelo G, Chaerkady R, Yu W, Hizal DB, Hess S, Zhao W, et al. Statistical models for the analysis of isobaric tags multiplexed quantitative proteomics. Journal of proteome research. ACS Publications; 2017;16:3124–36.

6. Kammers K, Cole RN, Tiengwe C, Ruczinski I. Detecting significant changes in protein abundance. EuPA open proteomics. Elsevier; 2015;7:11–9.

7. Pursiheimo A, Vehmas AP, Afzal S, Suomi T, Chand T, Strauss L, et al. Optimization of statistical methods impact on quantitative proteomics data. Journal of proteome research. ACS Publications; 2015;14:4118–26.

8. Schwenk JM, Omenn GS, Sun Z, Campbell DS, Baker MS, Overall CM, et al. The human plasma proteome draft of 2017: Building on the human plasma peptideatlas from mass spectrometry and complementary assays. Journal of proteome research. ACS Publications; 2017;16:4299–310.

9. Zahn-Zabal M, Michel P-A, Gateau A, Nikitin F, Schaeffer M, Audot E, et al. The neXtProt knowledgebase in 2020: Data, tools and usability improvements. Nucleic Acids Research. Oxford University Press; 2020;48:D328–34.

10. Goeman JJ, Buhlmann P. Analyzing gene expression data in terms of gene sets: Methodological issues. Bioinformatics. Oxford University Press; 2007;23:980–7.

11. Hassis ME, Niles RK, Braten MN, Albertolle ME, Witkowska HE, Hubel CA, et al. Evaluating the effects of preanalytical variables on the stability of the human plasma proteome. Analytical biochemistry. Elsevier; 2015;478:14–22.

12. Kaisar M, Dullemen LF, Thézénas M-L, Akhtar MZ, Huang H, Rendel S, et al. Plasma degradome affected by variable storage of human blood. Clinical proteomics. BioMed Central; 2016;13:26.
